# Single‐cell RNA sequencing and high‐dimensional flow cytometry reveal distinct peripheral immune landscapes of type 1 autoimmune pancreatitis and pancreatic ductal adenocarcinoma

**DOI:** 10.1002/ctm2.70680

**Published:** 2026-04-21

**Authors:** Chenxiao Liu, Tianyi Che, Airu Liu, Jiaxin Wang, Qidi Yang, Yiwen Tu, Zonghao Liu, Xiaonan Shen, Xiangyi He, Tingting Gong, Ling Zhang, Zhengji Song, Junjie Fan, Yue Zeng, Wenbin Zou, Youqiong Ye, Yao Zhang, Minmin Zhang, Duowu Zou, Chunhua Zhou

**Affiliations:** ^1^ Department of Gastroenterology Ruijin Hospital, Shanghai Jiao Tong University School of Medicine Shanghai China; ^2^ Yunnan Digestive Endoscopy Clinical Medical Center Department of Gastroenterology The First People's Hospital of Yunnan Province Yunan China; ^3^ Department of Gastroenterology Shanghai General Hospital Shanghai Jiao Tong University School of Medicine Shanghai China; ^4^ Department of Gastroenterology Changhai Hospital, Naval Medical University Shanghai China; ^5^ Shanghai Institute of Immunology Department of Immunology and Microbiology Shanghai Jiao Tong University School of Medicine Shanghai China

**Keywords:** autoimmune pancreatitis, biomarker, diagnosis, pancreatic ductal adenocarcinoma, peripheral blood mononuclear cell, single‐cell RNA sequencing

## Abstract

**Background:**

Autoimmune pancreatitis (AIP) is a chronic pancreatic inflammatory disease that is often difficult to differentiate from pancreatic cancer. Some AIP patients may even progress into pancreatic ductal adenocarcinoma (PDAC). We sought to delineate the peripheral immunological landscape of AIP, identify its differences from PDAC and find novel biomarkers for disease differentiation.

**Methods:**

Single‐cell RNA/BCR sequencing (scRNA/BCR‐seq) was performed on peripheral blood mononuclear cells (PBMCs) from 10 type 1 AIP patients. Public PBMC sequencing data from 13 PDAC patients and 11 healthy volunteers were integrated in the analysis. Fourteen‐colour flow cytometry was conducted in independent cohorts for validation.

**Results:**

The analyses revealed a significantly higher proportion of IgG4high‐switched memory B cells in patients with AIP than in PDAC. These cells, characterised by high CD23 expression, exhibited enhanced antigen‐presenting capacity and might differentiate into pancreatic plasma cells in AIP. Compared with PDAC, AIP was characterised by an increased frequency of T follicular helper (Tfh) cells with a more pronounced exhaustion‐like phenotype. Coculture experiments demonstrated that IgG4high‐switched memory B cells can promote Tfh cell differentiation through major histocompatibility complex‐mediated antigen presentation. *TREM2*‐up‐regulated intermediate monocytes were also increased in AIP and showed greater potential to differentiate into macrophages. The Boruta algorithm identified proportional changes in these subsets as useful for disease differentiation, and these findings were validated by multi‐colour flow cytometry. A nomogram was established, with an AUC of .94 in the internal cohort and .88 in the external cohort. As for prognostic prediction, the reduction rate of Tfh cells after steroid therapy was associated with relapse risk.

**Conclusion:**

By integrating scRNA/BCR‐seq and flow cytometry, we identified three novel immune cell subsets in PBMCs of AIP patients and confirmed their diagnostic and prognostic value. A flow cytometry‐derived nomogram based on these subsets provides a novel tool for differentiating patients with AIP from those with PDAC.

## Graphical abstract

Research Highlights

• Illustrated the single‐cell peripheral immune landscape of AIP and its distinction from PDAC.

• Identified expanded CD23^+^IgG4high‐switched memory B cells, Tfh cells and enhanced antigen‐presentation interactions between them.

• Multi‐centre flow cytometry validated a nomogram incorporating these subsets for differential diagnosis.

• Tfh‐cell decrease rate after steroid therapy was associated with relapse risk in AIP.

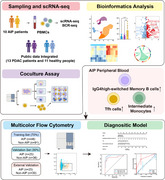

## 1 BACKGROUND

Autoimmune pancreatitis (AIP) is a unique type of chronic pancreatitis (CP), predominantly affecting elderly men and is characterised by a favourable response to steroid therapy.^1^, ^2^ Type 1 AIP is also recognised as the pancreatic manifestation of IgG4‐related disease (IgG4‐RD), a systemic autoimmune disorder.^3^ AIP often presents acutely but frequently progresses to late‐stage complications,^4^ which notably impair patients’ life quality, highlighting the need for accurate early diagnosis of AIP. Although serum IgG4 has long served as a diagnostic biomarker,^1^ its sensitivity and specificity are limited, particularly in distinguishing AIP from pancreatic ductal adenocarcinoma (PDAC).^5^ Moreover, the pathogenic role of IgG4 remains unclear and is insufficient to fully explain the variability in treatment responses, leaving a gap in our understanding of the disease‐driving cellular subsets.^6^

These limitations highlight the need for a deeper exploration of AIP pathogenesis, particularly its cellular contributors. Previous studies reported distinct immune cells to play critical roles in IgG4‐RD and AIP development, including plasmablasts,^7^ T follicular helper (Tfh) cells,^3^ cytotoxic T cells^8^, ^9^ and macrophages.^10^ However, these studies are limited in providing an overview of immunological changes in AIP and comparing these changes with those of other fibroinflammatory or malignant diseases. Single‐cell RNA sequencing (scRNA‐seq) made it possible to overcome some of these limitations. For example, Munemura et al. used scRNA‐seq to identify two disease‐specific Tfh cell subtypes in IgG4‐RD and Kimura disease.^11^ Moreover, by combining scRNA‐seq with spatial transcriptomic sequencing, our team recently reported the expansion of age‐associated B cells (ABCs) in pancreas of patients with AIP, whereas they were not detected in the pancreas of patients with CP.^12^ However, the peripheral immune landscape of AIP patients and its differences from that of PDAC remain incompletely understood, and translating these findings into clinically practical biomarkers remain challenging.

The intricate relationship and shared features between AIP and PDAC further amplify the clinical significance of this research. Diagnostic confusion often arises because of their overlapping symptoms and inappropriate steroid administration may potentially exacerbate PDAC progression, with devastating consequences.^4^ Additionally, patients with IgG4‐RD and AIP appear to have an increased susceptibility to cancer, particularly pancreatobiliary cancers, whereas steroid therapy may mitigate this risk.^13^, ^14^ These observations underscore the need to characterise the immune differences between AIP and PDAC.

In this study, we performed scRNA‐seq and single‐cell B‐cell receptor repertoire sequencing (scBCR‐seq) on peripheral blood mononuclear cells (PBMCs) from 10 patients with AIP. We also incorporated previously published scRNA‐seq data from pancreatic cells of patients with AIP^12^ as well as PBMC data from patients with PDAC^15^ and healthy controls (HCs).^16^ Using a 14‐colour flow cytometry panel, we validated the changes in these subsets and translated these findings into a clinically applicable nomogram. Collectively, our work characterises single‐cell‐level immunological alterations in AIP and identifies potential biomarkers for diagnosis and prognostic assessment.

## 2 METHODS

### 2.1 Patient samples

Peripheral whole‐blood samples were collected from 10 patients with AIP at Ruijin Hospital (Table S1). These samples were used for scRNA‐seq and scBCR‐seq. All enrolled patients met the 2011 International Consensus Diagnostic Criteria (ICDC).^1^

For flow cytometry analysis, we established two independent cohorts. Internal cohort: Samples were collected at Ruijin Hospital between 2021 and 2025. This cohort included 71 with AIP patients (excluding those in the sequencing cohort described above), 39 patients with CP, 45 patients with PDAC and 43 healthy volunteers (Tables S2 and S3). External cohort: Samples were collected at Changhai Hospital and Shanghai General Hospital between 2024 and 2026. This cohort included 25 patients with AIP, 10 patients with CP, 10 patients with PDAC and 10 healthy volunteers (Tables S2 and S3). All samples were stored under the same conditions, thawed simultaneously and processed using a standardised experimental workflow. In addition, the flow cytometry procedures were performed in a consistent manner across all samples to minimise technical variability and reduce potential batch effects to the greatest extent possible.

### 2.2 ScRNA‐seq of PBMCs

PBMCs were isolated following the Ficoll overlay method.^17^ The libraries were then prepared using 10x Chromium Next GEM Single Cell 5′ Kit v2 (10X Genomics). The resulting count matrices were imported into R statistical software (4.4.0). All analysis were conducted with Seurat (5.1.3).^18^

### 2.3 Public data acquisition

scRNA‐seq data of pancreatic cells in AIP were retrieved from the Genome Sequence Archive (GSA) with accession number HRA007090.^12^ This dataset includes scRNA‐seq and scBCR‐seq data of biopsy lesion tissue samples from 10 patients with type 1 AIP and eight non‐inflamed controls at Ruijin Hospital. We incorporated scRNA‐seq data of the 10 AIP patients, eight of whom were the same individuals who underwent PBMC scRNA‐seq in our study.

scRNA‐seq data of PBMCs in PDAC were retrieved from the GSA with accession number HRA003065.^15^ The dataset contains scRNA‐seq data from PBMC and tumour samples collected from 28 patients with PDAC, before and after AG treatment. We incorporated PBMC scRNA‐seq data of 13 patients who had not received prior treatment.

scRNA‐seq data of PBMCs from HCs were retrieved from the Gene Expression Omnibus with accession number GSE165080.^16^ This dataset includes scRNA‐seq data of 53 samples (42 from patients with COVID‐19 and 11 from HCs). We incorporated scRNA‐seq data of 11 HCs from this dataset. All public datasets were subjected to the same quality‐control procedures as the scRNA‐seq data in our study, as described below.

### 2.4 Cell demultiplexing and quality control

Genes expressed in fewer than three cells were removed. Cells were filtered out if they had more than 10% mitochondrial gene expression and more than 25% red cell gene expression. Cells with 500–50 000 UMI counts and 200 to 7000 genes were retained. In addition, doublets were removed by Scrublet (0.2.3).^19^

### 2.5 Normalisation and dataset combination

‘NormalizeData’ function was used for data normalisation. Seurat objects were merged using Seurat (5.1.3). To compute integration anchors, the ‘SelectIntegrationFeature’ and ‘FindIntegrationAnchors’ functions were employed. These anchors rely on integration features, defined as genes that exhibit consistent variability across all datasets involved in anchor establishment. After anchor calculation, ‘IntegrateData’ function was applied to merge distinct datasets. Batch‐effect correction was performed on the integrated object using Harmony (1.2.0).^20^ Specifically, ‘RunHarmony’ function was utilised to reconcile variances.

### 2.6 Cell clustering and cell type annotation

Top 4000 most variable genes were identified by ‘FindVariableFeatures’ function. ‘FindNeighbors’ and ‘FindClusters’ functions were used to for graph clustering. Uniform manifold approximation and projection (UMAP) was used for dimensionality reduction.

### 2.7 Differential gene expression analysis

‘FindAllMarkers’ function was used to identify differentially expressed genes (DEGs) between clusters with min.pct at .1 and logfc.threshold set at .25. ‘AggregateExpression’ was used for pseudobulk analysis. The ‘FindMarkers’ function was used to identify DEGs between disease groups. Full lists of marker genes were in Tables S4–10.

### 2.8 Functional enrichment analysis

GO biological process (GO BP) and reactome pathway analyses were performed using clusterProfiler (4.12.0).^21^ Gene set variation analysis (GSVA) was performed using the GSVA package (1.52.3) to calculate pathway activity scores and the exhaustion score of Tfh cells in different disease groups. GSVA was run using the ‘gsvaParam’ function with kcdf = ‘Poisson’, minSize = 1 and maxSize = Inf. The relevant signature genes were listed in Table S11.

### 2.9 ScBCR‐seq analysis

BCR clonotypes were assigned with the Cell Ranger V(D)J pipeline (v5.0.1; 10x Genomics; GRCh38), yielding clonotype frequencies and barcode annotations. Productive BCR contigs were assigned to individual cells according to unique cell barcodes, and clonotype information, including clonotype frequency, barcode annotation and CDR3 sequence features, was extracted. Subsequent analyses was conducted using the scRepertoire package (2.0.7).^22^

### 2.10 Cellular communication analysis

We use CellChat (1.6.2) to analyse intercellular communication through ligand‐receptor pairs.^23^ CellchatDB.human was used as the database for signalling pathways. Communication probabilities were calculated using the truncated mean method with trim = 0.1 and population.size = TRUE, and interactions involving fewer than three cells were filtered out.

### 2.11 Trajectory analysis

Developmental trajectory analysis was performed using Monocle3 (v2.22.0).^24^ Highly variable genes were selected, followed by dimensionality reduction and cell ordering for pseudotime inference. Trajectory graphs were learned after UMAP embedding, and pseudotime‐related genes were identified using ‘graph_test’.

### 2.12 Boruta feature selection analysis

The Boruta (v8.0.0)^25^ was used for selection. Specifically, the model was fitted using the ‘Boruta’ function with diagnosis as the outcome and all candidate variables as predictors. Relevant features were then extracted using the ‘getSelectedAttributes’ function with tentative attributes retained. Feature‐level importance statistics and selection decisions were obtained using the ‘attStats’ function, which provided summary measures of variable importance and the final Boruta classification (confirmed, tentative or rejected). The distributions of importance scores for retained variables were further visualised using ridge plots based on the *z*‐score distributions of feature importance.

### 2.13 Flow cytometry

Flow cytometry was performed as in our previous study^12^ and detailed procedures are provided in the Supporting Information.

### 2.14 Isolation of cells, transfection and co‐culture assay

B cells and Tfh cells were isolated from the PBMCs of patients with AIP using a FACSAria III cell sorter (BD Biosciences). Purified CD19^+^B cells and PD‐1^+^CD4^+^Tfh cells were separately seeded in 96‐well round‐bottom plates as in our previous study.^12^

Before transfection, B cells were stimulated with 1 µg/mL CD40L (PeproTech; 310‐02‐10UG) and 10 ng/mL IL‐4 (PeproTech; 200‐04‐5UG) for 24 h. Negative control small interfering RNA (siRNA) or human leukocyte antigen‐DR isotype (HLA‐DR)‐targeting siRNA (Table 1) was prepared and mixed with CALNP™ RNAi in vitro (D‐Nano Therapeutics; DN001‐01). After siRNA transfection mixture added, B cells were incubated for an additional 24 h before co‐culture. Tfh cells were incubated with CD3/CD28 beads (EasyIso; AH2001). After 48 h they were then co‐cultured with B cells in the presence of 10 ng/mL tetanus toxoid (TT) (MeilunBio; MB12003) for 3 days. Cells and culture supernatants were subsequently collected for experiments described below.

**TABLE 1:** siRNA sequences.

siRNA	Forward primer (5′→3′)	Reverse primer (5′→3′)
si‐Negative control	UUCUCCGAACGUGUCACGUTT	ACGUGACACGUUCGGAGAATT
si‐HLA‐DRA	GGGAAGACCACCUUUUCCGTT	CGGAAAAGGUGGUCUUCCCTT

### 2.15 Reverse transcription quantitative polymerase chain reaction

Reverse transcription quantitative polymerase chain reaction (RT‐qPCR) was performed as in our previous study^12^ and detailed procedures are provided in the Supporting Information. Primers were provided in Table 2.

**TABLE 2:** qPCR primer sequences.

Gene	Forward primer (5′→3′)	Reverse primer (5′→3′)
*HLA‐DRA*	AGTCCCTGTGCTAGGATTTTTCA	ACATAAACTCGCCTGATTGGTC
*GAPDH*	ACAACTTTGGTATCGTGGAAGG	GCCATCACGCCACAGTTTC
*ICOS*	ACAACTTGGACCATTCTCATGC	TGCACATCCTATGGGTAACCA
*CXCR5*	GGTCACCCTACCACATCGTC	GCCATTCAGCTTGCAGGTATTG
*BCL6*	ACACATCTCGGCTCAATTTGC	AGTGTCCACAACATGCTCCAT

### 2.16 Enzyme‐linked immunosorbent assay (ELISA)

Briefly, 50 µL of cell culture supernatant from each co‐culture well was used for measurement of IL‐21 using a human IL‐21 ELISA kit (Mlbio; ml027406). A total of 450 nm absorbance was measured in Infinite 200 PRO microplate reader (TECAN).

### 2.17 Diagnostic model construction and validation

Patients in the internal cohort were randomly divided into a training set (70%) and an internal validation set (30%) after stratification by diagnostic group. Univariable logistic regression was first performed in the training set. Variables selected on the basis of univariable significance (*p* < .05) were then entered into a multi‐variable logistic regression model. Multi‐collinearity among variables in the multi‐variable model was assessed using the variance inflation factor. A nomogram was generated based on the final model, with predicted probabilities displayed at .1, .3, .5, .7 and .9. Methodological details regarding model performance are provided in the Supporting Information.

### 2.18 Prognostic cohort

For prognostic evaluation, whole‐blood samples were collected from patients with AIP at diagnosis, during steroid induction therapy (1–3 months after starting treatment) and during maintenance therapy (>3 months after treatment initiation). Relapse was defined by recurrent symptoms and/or new or recurrent radiologic findings suggestive of disease activity. The rate of decrease was calculated as the difference between two measurements divided by the time interval in days, according to a previously published method.^26^

### 2.19 Statistical analysis

Statistical methods used included two‐tailed *t*‐tests, paired *t*‐test, Mann–Whitney *U*‐test, Fisher's exact test and one/two‐way analysis of variance (ANOVA), as specified in the figure legends and Supporting Information. For multiple comparisons, Bonferroni correction and Dunnett's multiple comparisons test were applied. All analyses were conducted In R or GraphPad Prism 6.0.

## 3 RESULTS

### 3.1 Single‐cell transcriptome profiling of PBMCs in patients with AIP

**FIGURE 1:**
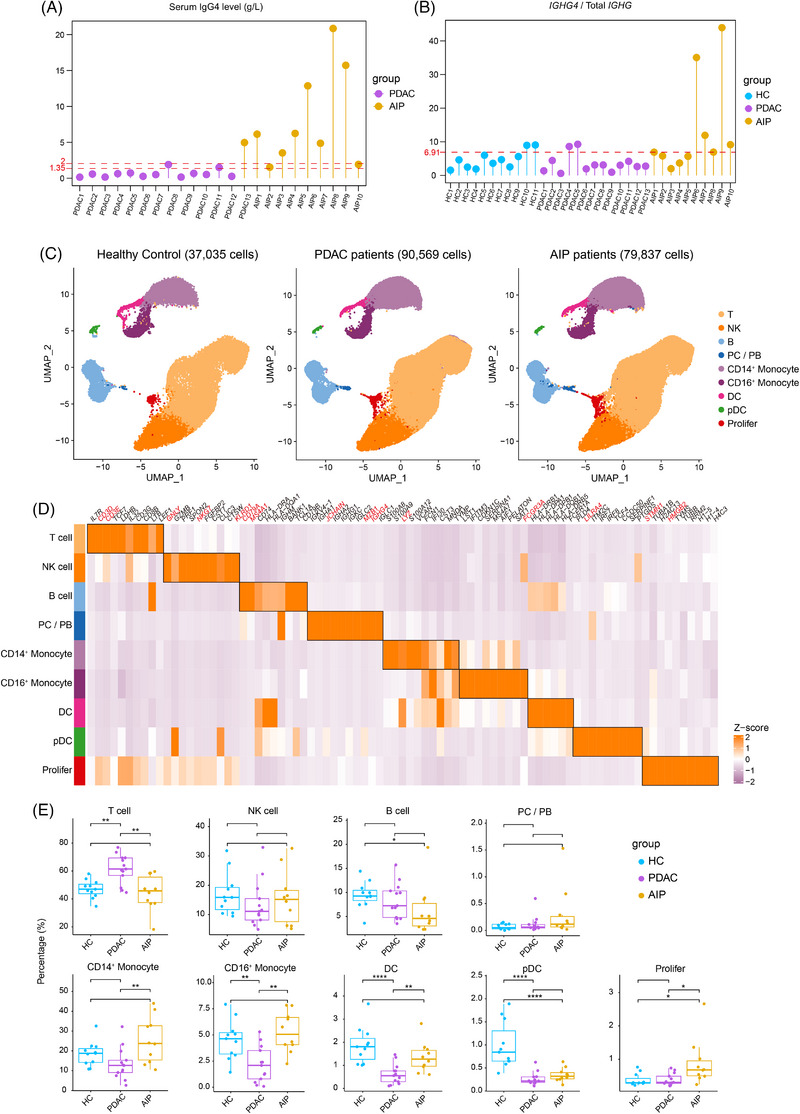
IgG4 levels and single‐cell cellular atlas of PBMCs in HCs, PDAC and AIP patients. (A) Lollipop plot showing serum IgG4 levels of PDAC patients (*n* = 13) and AIP (*n* = 10) patients included in scRNA‐seq analysis. The upper red dotted line indicates the upper limit of the normal serum IgG4 value at our institution. The lower red dotted line denotes the 135 mg/dL threshold, which serves as the criterion for defining elevated serum IgG4 levels in the JPS2018. (B) Lollipop plot showing the RNA expression level of *IGHG4* gene relative to total *IGHG* transcripts in our scRNA‐seq dataset. The red dotted line indicates the medium value of *IGHG4* gene expression relative to total *IGHG* expression in AIP patients. (C) UMAP plots showing nine major cell clusters from PBMCs of HCs (*n* = 11), PDAC patients (*n* = 13) and AIP (*n* = 10) patients in different colours. (D) Heatmap showing the relative expression (*z* score) of top 10 marker genes of indicated major cell clusters. Clusters are coloured as in (C). (E) Boxplots showing the comparison of the percentage of nine major cell clusters in PBMCs of HCs, PDAC patients and AIP patients. Statistical differences were determined by Mann–Whitney *U*‐test (*p < .05, **p < .01).

PBMCs were collected from patients with AIP (AIP1‐10) and PDAC (PDAC1‐13) (Figure S1A), and all blood samples were obtained before the initiation of therapy. Using a cutoff value of 1.35 g/L, two patients with PDAC showed elevated serum IgG4 levels. Conversely, with a cutoff of 2.00 g/L, two patients with AIP exhibited normal serum IgG4 levels (Figure 1A,B). The two serum IgG4 cutoff values were selected based on the two major consensus criteria for AIP: 1.35 g/L, as defined by the Japanese Pancreas Society (JPS) criteria,^27^ and 2.00 g/L, corresponding to the upper limit of normal in our local laboratory and consistent with the ICDC.^1^ These findings suggest that serum IgG4 alone lacks sufficient sensitivity and specificity. The complete clinical information of all patients is detailed in Table S1. Initially, we analysed the IGHG4/IGHG expression ratio in each patient and found that some HCs and PDAC patients had values higher than the median value in patients with AIP. Following the standard workflow, 37 035 cells from HCs, 90 569 from patients with PDAC and 79 837 from patients with AIP were retained (Figures 1C and S1B,C). Using established marker genes, we identified nine primary immune cell types, which were visualised via UMAP embeddings (Figures 1C,D and S1D,E). Compared with HCs, AIP patients showed significantly decreased proportions of B cells and plasmacytoid dendritic cells, as well as a significantly increased proportion of *MKI67* ^+^ proliferating cells (Figure 1E).

### 3.2 Diversity of B cell and memory B cell subsets

**FIGURE 2:**
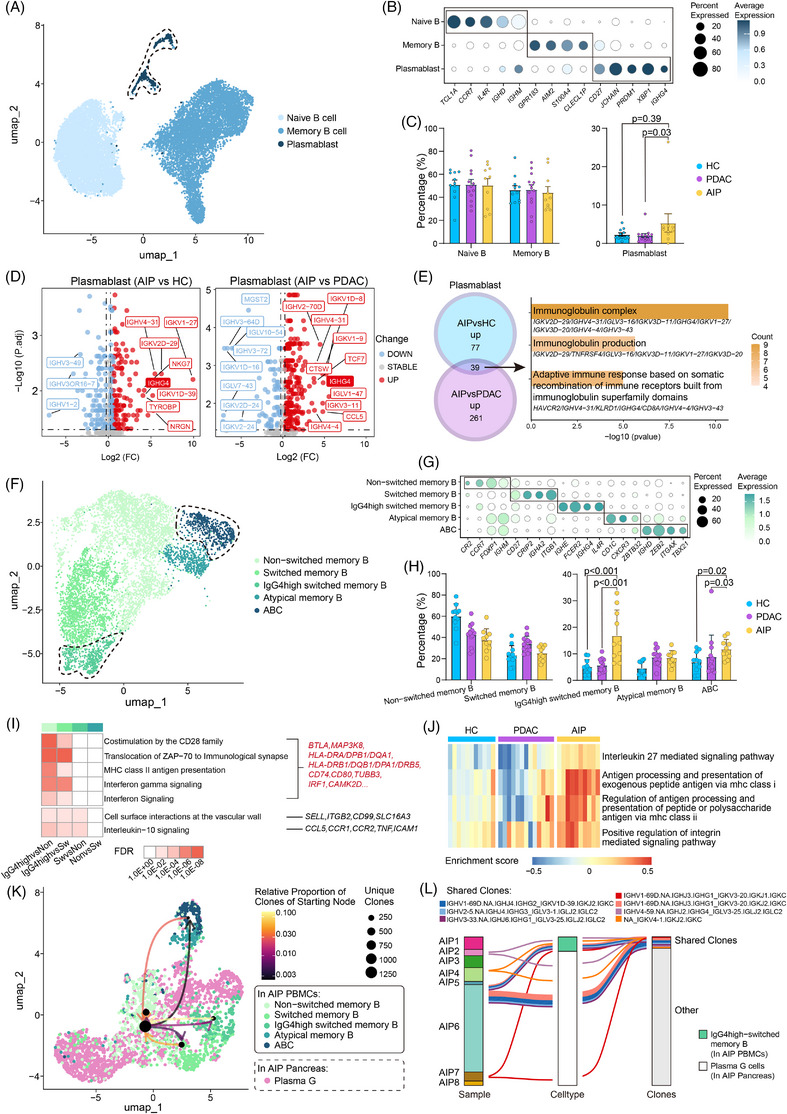
Clustering of peripheral B cell subsets and memory B cell subsets, and functional and BCR analysis of these subsets in HCs, PDAC patients and AIP patients. (A) UMAP plot showing three B cell subsets from PBMCs of HCs, PDAC patients and AIP patients in different colours. (B) Dot plot displaying representative markers in each B cell subsets. (C) Bar plots comparing the percentage of B cell subsets. Statistical differences were determined by Mann–Whitney *U*‐test. (D) Volcano plot showing the up‐regulated and down‐regulated DEGs in the plasmablast subset, derived from pseudobulking analysis. (E) Venn plot illustrating the overlap of up‐regulated DEGs. Representative enriched terms from the GO BP analysis of these common DEGs in are presented in the right. (F) UMAP plot showing five memory B cell subsets from PBMCs of HCs, PDAC patients and AIP patients in different colours. (G) Dot plot displaying representative markers in each memory B cell subsets. (H) Bar plots comparing the percentage of memory B cell subsets. Statistical differences were determined by Mann–Whitney *U*‐test. (I) Heatmap showing the enriched Reactome pathways of DEGs among IgG4high‐switched memory B cells, switched memory B cells and non‐switched memory B cells. (J) Heatmap displaying the GSVA enrichment scores across different pathways in IgG4‐high switched memory B cells from HCs, PDAC patients and AIP patients. (K) Weighted and oriented network plot combined with UMAP plots showing the clonotype connection among five memory B cell subsets in the PBMCs of AIP patients and plasma G cells in the pancreas of AIP patients. (L) Alluvial plot tracking specific clones from IgG4high‐switched memory B cells (PBMCs) to pancreatic plasma cells in AIP patients (shared clones in the same patient coloured identically).

To characterise variations in B‐cell subtypes among the disease groups, we first categorised B cells into three primary subsets based on classical marker genes (Figures 2A,B and S2A). Genes highly expressed in each subset are shown in Figure S2B. In the AIP group, plasmablasts showed a significantly increased proportion (Figures 2C and S2C). We performed pseudobulking on these cells, an approach that substantially reduces false positive gene associations with different disease conditions (Figure S2D). The analysis showed that specific up‐regulation of *IGHG4* in patients with AIP compared with HCs and patients with PDAC (Figure 2D). GO BP analysis showed enrichment of processes related to the immunoglobulin complex (Figure 2E).

In subsequent analyses, we subclustered memory B cell subsets into five distinct populations (Figures 2F,G and S2E,F). A significant increase in IgG4high‐switched memory B cells was observed in AIP (Figures 2H and S2G). They showed relatively high expression of *IGHE*, *IGHG4* and *FCER2*, which encodes CD23. This expression pattern suggested that CD23 may serve as a useful surface marker for the flow cytometric identification of IgG4high‐switched memory B cells. The results also showed that ABCs in were increased in AIP PBMCs, with a predominant IgD^+^ phenotype (Figure 2G,H), whereas our previous findings suggested that enriched ABCs within the pancreatic parenchyma predominantly exhibited an IgD^−^ phenotype.^12^

Enriched pathways in IgG4high‐switched memory B cells included costimulation via the CD28 family, major histocompatibility complex (MHC) class II antigen presentation and interferon‐γ (IFN‐γ) signalling (Figure 2I), which may indicate that these cells have antigen‐presenting properties and may interact actively with T cells. Pseudobulking differential expression analysis showed that *AICDA* (also known as AID) was exclusively up‐regulated in IgG4high‐switched memory B cells from AIP patients (Figure S2H–J), which may suggest that these cells are undergoing active antibody class switching, recombination and somatic hypermutation in AIP. GSVA analysis showed enrichment of pathways involving regulation of antigen processing and presentation via MHC II and MHC I (Figure 2L), which is possibly consistent with an enhanced antigen‐presenting capacity of IgG4high‐switched memory B cells. Furthermore, IL‐27 signalling was also increased in these cells (Figure 2L), which may be relevant because IL‐27 can induce the expression of AICDA and MHC II.

We next explored the potential differentiation of peripheral blood memory B cells into plasma cells within the pancreas of AIP patients, integrating scRNA‐seq and scBCR‐seq data from PBMCs and pancreatic cells.^12^ Our results showed that IgG4high‐switched memory B cells exhibited the greatest degree of clonotype overlap with pancreatic plasma cells (Figure 2K). Notably, we identified seven unique clonotypes in IgG4high‐switched memory B cells that were also detected in the plasma cells of the same AIP patient (Figure 2L). These results may suggest that IgG4high‐switched memory B cells have the potential to differentiate into plasma cells in AIP and may represent a possible source of pancreatic autoantibodies.

### 3.3 Characterisation of CD4^+^ T cell subsets and their crosstalk with IgG4high‐switched memory B cells

**FIGURE 3:**
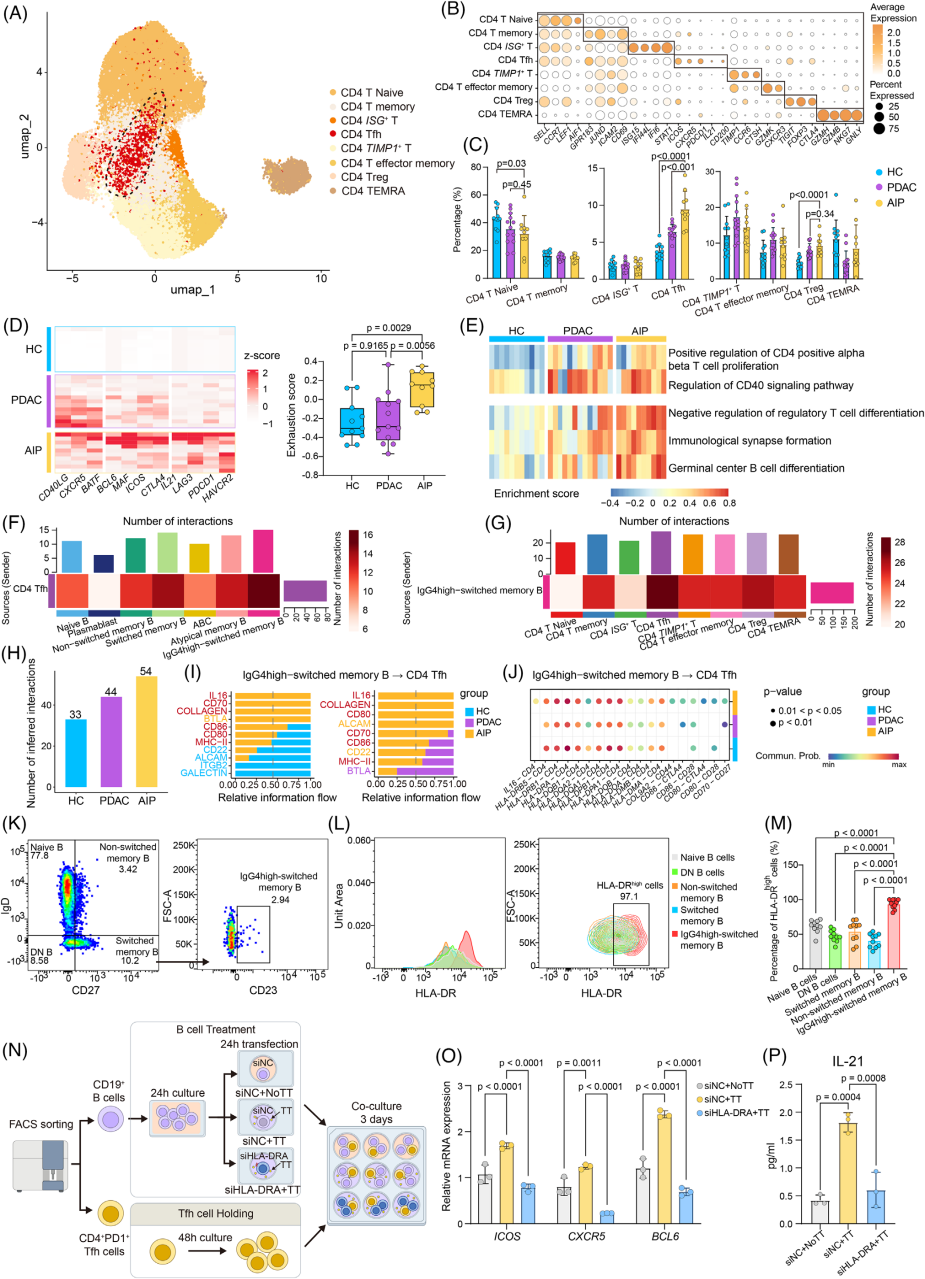
Heterogeneity of peripheral CD4^+^ T cells and their interactions with B cell subsets in HCs, PDAC patients and AIP patients. (A) UMAP plot showing 8 CD4**^+^** T cell subsets from PBMCs of HCs, PDAC patients and AIP patients in different colours. (B) Dot plot displaying representative markers in each CD4^+^ T‐cell subset. (C) Bar plots comparing the percentage of CD4**^+^** T cell subsets. Statistical differences were determined by Mann–Whitney *U*‐test. (D) (left) Heatmap showing the relative expression (*z* score) of genes in the CD4^+^ Tfh subset of each individual subject. (Right) Boxplot showing the comparison of CD4^+^ Tfh cell exhaustion scores among individual subjects. Statistical differences were determined by Mann–Whitney *U*‐test. (E) Heatmap displaying the GSVA enrichment scores across different pathways in CD4^+^ Tfh cells from HCs, PDAC patients and AIP patients. (F) Heatmap showing the number of interactions from CD4**^+^** Tfh cells (sender) to all B cell and memory B cell subsets (receivers). (G) Heatmap showing the number of interactions from IgG4high‐switched memory B cells (sender) to all CD4**^+^** T cell subsets (receivers). (H) Bar plot showing the overall number of interactions between IgG4high‐switched memory B cells and CD4**^+^** Tfh cells in HCs, PDAC patients and AIP patients. (I) Bar plot showing the information flow from IgG4high‐switched memory B cells to CD4**^+^** Tfh cells. (J) Dot plot illustrating the specific ligand‒receptor communication probabilities. (K) Representative flow cytometric gating strategy for B‐cell subsets, including naive B cells, double‐negative (DN) B cells, non‐switched memory B cells, switched memory B cells and IgG4 high‐switched memory B cells. (L) Representative flow cytometry plots showing HLA‐DR expression in different B‐cell subsets. (M) Comparison of the percentage of HLA‐DR^high^ cells among different B‐cell subsets. Statistical differences were determined by one‐way ANOVA followed by Dunnett's multiple comparisons test. (N) Schematic diagram of the co‐culture experiment. CD19^+^ B cells were transfected with siRNA (siNC) or HLA‐DRA siRNA (si HLA‐DRA) and co‐cultured with Tfh cells. Human model antigen tetanus toxoid (TT) stimulation was applied as indicated. (O) Relative mRNA expression of *ICOS*, *CXCR5* and *BCL6* in Tfh cells after co‐culture under different treatment conditions. Statistical differences were determined by two‐way ANOVA followed by Dunnett's multiple comparisons test. (P) IL‐21 levels in the culture supernatant under different treatment conditions. Statistical differences were determined by one‐way ANOVA followed by Dunnett's multiple comparisons test.

To investigate the dynamic changes in various T cell subtypes, we initially focused on CD4^+^ T cells and identified eight distinct subsets based on subtype markers (Figures 3A,B and S3A,B). Among CD4^+^ T cells, CD4 naïve T cells showed a declining trend, while Tfh cells were significantly increased in AIP patients (Figures 3C and S3C). Treg (Regulatory T) cells were increased in AIP patients relative to HCs but exhibited no significant difference when compared with PDAC patients (Figures 3C and S3C).

Pseudobulk differential gene analysis revealed that *LAG3* was one of the top DEGs between AIP and the other two groups (Figure S3D,E). *LAG3* ^+^ Tfh cells have previously been reported to be abundant in the inflamed tissues of patients with IgG4‐RD.^11^ We next examined genes associated with Tfh cell function. The results showed coordinated up‐regulation of *BCL6* alongside *MAF* and *ICOS* which may suggest stronger Tfh cell differentiation in AIP compared with PDAC and HCs (Figure 3D). Notably, exhaustion‐related markers and exhaustion‐associated scores were preferentially increased in Tfh cells from AIP patients (Figure 3D), which may be consistent with the presence of an exhaustion‐like transcriptional/phenotypic state that may reflect chronic Tfh‐cell activation in AIP. Additionally, IL‐21, a cytokine reported to facilitate B cell differentiation and is highly secreted by *LAG3* ^+^ Tfh cells in IgG4‐RD,^12^ was exclusively up‐regulated in AIP patients (Figure 3D). We also noted that *CTLA4* was up‐regulated in AIP Tfh cells despite the absence of *FOXP3* expression (Figure 3D). Consistent with this, GSVA analysis showed enrichment of ‘negative regulation of regulatory T cell differentiation’ in AIP Tfh cells (Figure 3E), which is possibly consistent with these cells being distinct from *FOXP3* ^+^ Treg cells or *FOXP3* ^+^ regulatory Tfh cells.

T peripheral helper (Tph) cells are a recently described subset in autoimmune diseases.^28^, ^29^ They serve as counterparts of Tfh cells and can migrate to inflamed sites in the absence of *CXCR5* expression.^29^ Therefore, we defined three phenotypically similar subsets (Figure S3F,G). Our results showed that CXCR5^low^PD1^high^ Tph cells are highly expanded in the peripheral blood of AIP patients (Figure S3F). Compared with Tfh cells, these Tph cells expressed higher levels of *CXCR3*, *CCR6*, *GATA3*, *LAG3* and *CTLA4*, but lower levels of *BCL6* and *IL‐21* (Figure S3H), which may suggest that they are more functionally similar to Tfh2 cells and Tregs than to germinal centre (GC) Tfh cells.^3^ However, given that these subsets are not independent clusters, a finding that may indicate transcriptomic similarity, we still classified them as a single subset ‘CD4^+^ Tfh cells’ in subsequent analyses.

We subsequently performed CellChat analysis to characterise interactions between Tfh cells and B cell lineages. Tfh cells was predicted to have the strongest inferred interactions with IgG4high‐switched memory B cells (Figure 3F,G) and their interactions were increased in AIP (Figure 3H). CTLA4–CD86/CD80 were exclusively detected between Tfh cells and IgG4high‐switched memory B cells in AIP and PDAC patients (Figure S3I), which may suggest that Tfh cells participate in constraining excessive B‐cell responses in AIP. Notably, multiple MHC II‐related signalling pathways were enriched between IgG4high‐switched memory B cells and Tfh cells in AIP (Figure 3I,G). This is consistent with our earlier observation that IgG4high‐switched memory B cells may have antigen‐presenting features. To further examine this possibility, we performed additional siRNA‐mediated functional experiments. We first confirmed by flow cytometry that the CD23‐identified IgG4high‐switched memory B cells expressed the highest level of HLA‐DR (Figure 3K–M). We then isolated CD19^+^B cells and CD4^+^PD‐1^+^Tfh cells from AIP patients, transfected B cells with HLA‐DRA‐specific siRNA or negative control siRNA and performed co‐culture experiments in the presence of the model antigen TT (Figures 3N and S3J). qPCR confirmed efficient and specific knockdown of HLA‐DRA mRNA expression in B cells (Figure S3K,L). TT stimulation increased the mRNA levels of core Tfh differentiation markers and effector molecules (*CXCR5*, *ICOS* and *BCL6*), whereas these effects were attenuated after HLA‐DRA knockdown in B cells (Figure 3O). ELISA analysis of culture supernatants showed a consistent trend in which TT stimulation increased IL‐21 secretion by Tfh cells, whereas this increase was reduced following HLA‐DRA knockdown in B cells (Figure 3P). These findings provide functional support for the possibility that IgG4high‐switched memory B cells can promote Tfh‐cell differentiation through HLA‐DRA‐dependent antigen presentation.

### 3.4 Characterisation of CD8^+^ T cell and natural killer cell subsets

We further subclustered CD8^+^ T cells and identified six subtypes (Figure S4A,B). Compared with HCs and patients with PDAC, CD8^+^ naïve T cells showed a declining trend in AIP, while CD8 KIR^+^ TEMRA cells tended to increase (Figure S4C).

CellChat analysis showed the strongest inferred interactions between CD8 KIR^+^ TEMRA cells and IgG4high‐switched memory B cells (Figure S4D). Specifically, IFNG–IFNGR1/IFNGR2 signalling was up‐regulated between these subsets in AIP (Figure S4E), which may suggest that CD8 KIR^+^ TEMRA cells are relatively important source of IFN‐γ for the activation of IgG4high‐switched memory B cells. Additionally, multiple MHC‐I signalling pathways were exclusively detected between IgG4high‐switched memory B cells and CD8^+^ T cells in AIP (Figure S4E).

Natural killer (NK) cells were subclustered into three subsets (Figure S4F,G). While no NK subtype showed significant proportional differences across groups, CD56^bright^ NK cells tended to increase in AIP versus HCs and PDAC (Figure S4H).

### 3.5 Myeloid cell subtypes and the possible role of intermediate monocytes in AIP

**FIGURE 4:**
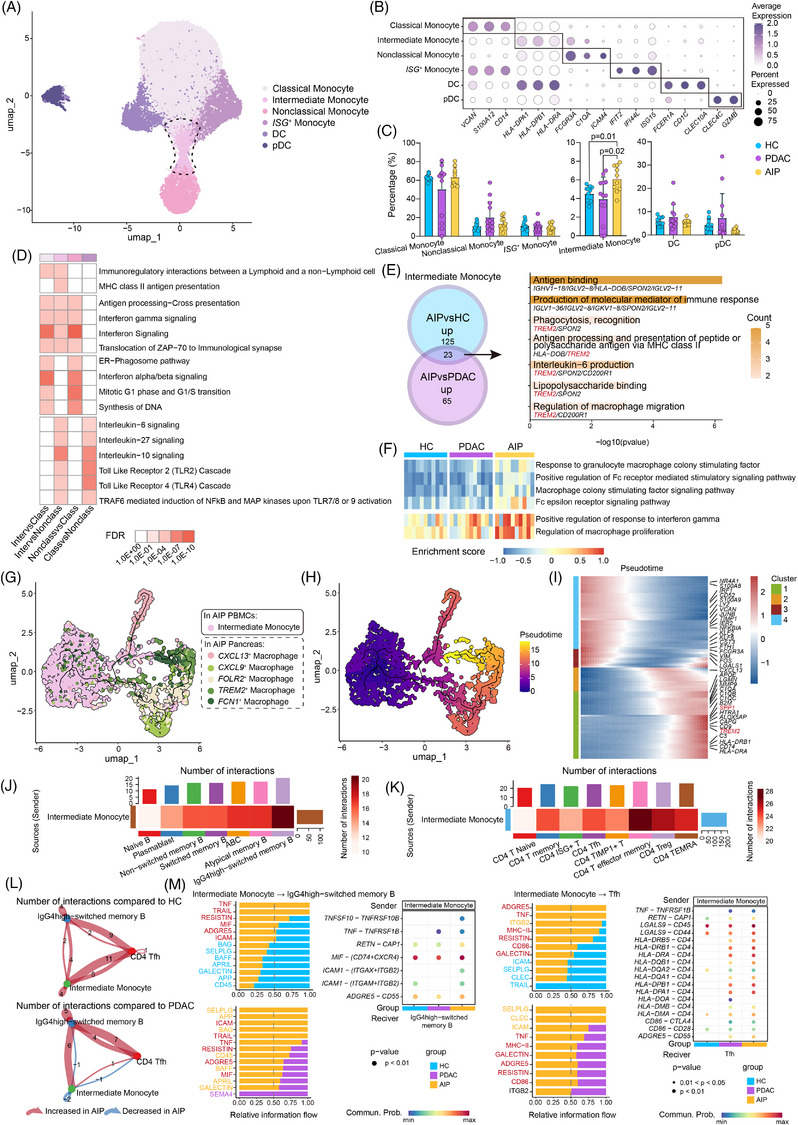
Distinct myeloid subsets, their differentiation trajectory and crosstalk with T/B cell subsets in HCs, PDAC and AIP Patients. (A) UMAP plot showing six myeloid cell subsets from PBMCs of HCs, PDAC patients and AIP patients in different colours. (B) Dot plot displaying representative markers in each myeloid cell subset. (C) Bar plots comparing the percentage of myeloid cell subsets. Statistical differences were determined by Mann–Whitney *U*‐test. (D) Heatmap showing the enriched reactome pathways of DEGs among classical monocytes, non‐classical monocytes and intermediate monocytes. (E) Venn plot illustrating the overlap of up‐regulated DEGs in intermediate monocytes. Representative enriched terms from the GO BP analysis of these common DEGs in intermediate monocytes are shown on the right. (F) Heatmap displaying the GSVA enrichment scores across different pathways in intermediate monocytes from HCs, PDAC patients and AIP patients. (G) UMAP plot showing intermediate monocytes in the PBMCs of AIP patients and five macrophage subsets in the pancreas of AIP patients in different colours. (H) UMAP plot showing the cells along with their trajectories. Colour represents the pseudotime of each cell. (I) Heatmap showing the dynamic expression (*z* score) changes of representative genes along the differentiation pseudotime of intermediate monocytes to macrophages. (J) Heatmap showing the number of interactions from intermediate monocytes (sender) to all B cell and memory B cell subsets (receivers). (K) Heatmap showing the number of interactions from intermediate monocytes (sender) to all CD4**^+^** T cell subsets (receivers). (L) Circle plot illustrating the relative number of interactions between IgG4‐high switched memory B cells, intermediate monocytes and CD4**^+^** Tfh cells in patients with AIP, compared with HCs and patients with PDAC. (M) (left) Bar plot showing the information flow from intermediate monocytes to IgG4high‐switched memory B cells. Right: Dot plot illustrating the specific ligand‒receptor communication probabilities. (Right) Bar plot showing the information flow from intermediate monocytes to CD4**^+^** Tfh cells. Right: Dot plot illustrating the specific ligand‒receptor communication probabilities.

Finally, to characterise the alterations in myeloid cells, we performed subclustering analysis of myeloid compartment. Six subtypes were identified according to their marker genes (Figures 4A,B and S5A,B). Intermediate monocytes, characterised by the expression of CD14, CD16 and HLA‐DR, were significantly increased in the PBMCs of AIP patients (Figures 4C and S5C).

Notably, intermediate monocytes enriched in immunoregulatory interactions between lymphoid and non‐lymphoid cells, as well as MHC II antigen presentation (Figure 4D). GO BP analysis was performed using the differentially up‐regulated DEGs specific to the AIP group and the results showed enrichment in BPs including phagocytosis, antigen processing and presentation and IL‐6 production, which may be related to the up‐regulation of *TREM2* (Figures 4E and S5D).

GSVA revealed that in intermediate monocytes from AIP patients, the response to macrophage colony‐stimulating factor, positive regulation in response to IFN‐γ and regulation of macrophage proliferation were significantly increased (Figure 4F). To further explore the differentiation of peripheral intermediate monocytes, we integrated scRNA‐seq data from PBMCs and pancreatic cells in AIP patients.^12^ Pseudotime trajectory analysis suggested that intermediate monocytes may differentiate into proinflammatory *CXCL13* ^+^ macrophages and *CXCL9* ^+^ macrophages and subsequently into anti‐inflammatory *TREM2* ^+^ macrophages and *FCN1* ^+^ macrophages (Figure 4G,H). This progression was marked by the up‐regulation of transcription factors, including *IRF1*, *KLF2*, *KLF6*, *NFKBIA* and *NR4A1*, which may serve as potential therapeutic targets (Figure 4I).

To investigate the cellular crosstalk involving intermediate monocytes, particularly with the expanded IgG4high‐switched memory B cells and Tfh cells, we performed CellChat analysis. The results showed that intermediate monocytes, acting as senders, had the most interactions with IgG4high‐switched memory B cells (Figures 4J,K and S5F). Compared with HCs, bidirectional interactions between these three subsets were increased in the AIP (Figure 4L). TNF–TNFRSF1B crosstalk was up‐regulated in the AIP group (Figure 4M), which may suggest that intermediate monocytes provide costimulatory signals that support B‐cell proliferation in AIP. Moreover, signalling pathways mediated by TNF, GALECTIN and MHC‐II were significantly up‐regulated in AIP group (Figure 4M). This finding may indicate that intermediate monocytes in AIP express multiple signalling molecules associated with inflammation responses and promote T‐cell activation.

### 3.6 Leveraging machine learning and multi‐colour flow cytometry to screen cell subsets for differential diagnosis

**FIGURE 5:**
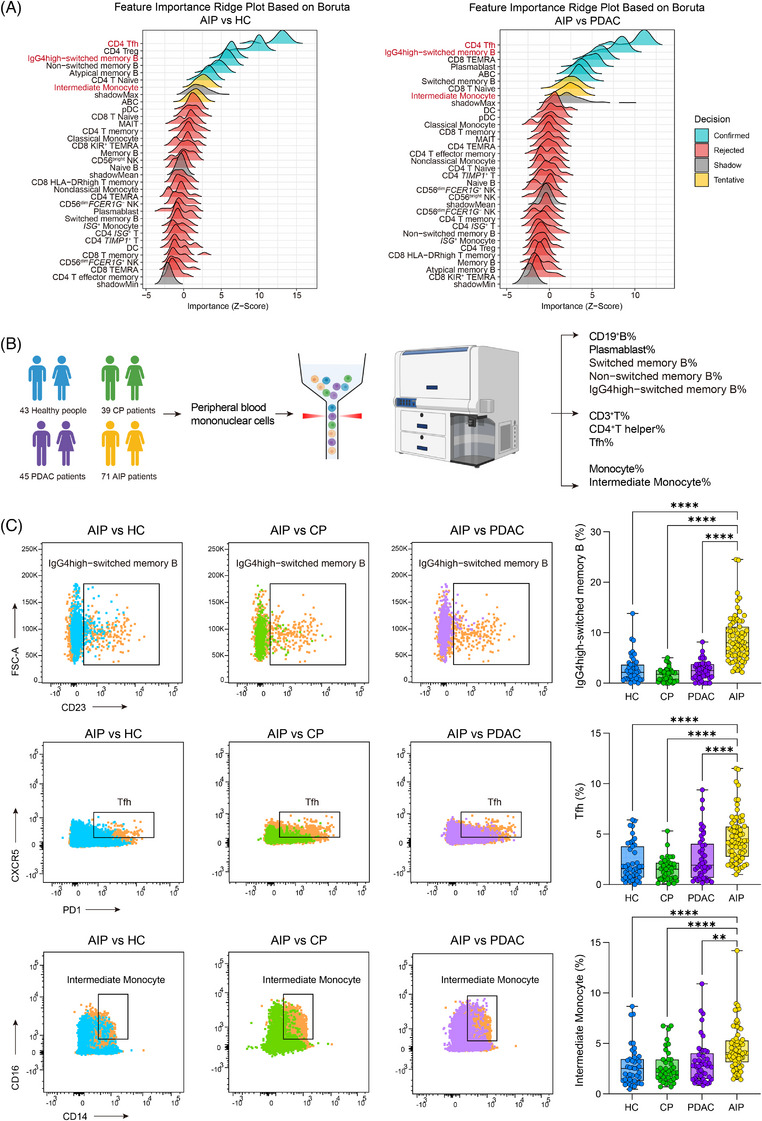
Screening of cell subsets for differential diagnosis using machine learning and multi‐colour flow cytometry. (A) Ridge plot illustrating the importance of all cell subsets identified in our scRNA‐seq analysis for differentiating between AIP and HCs, as well as between AIP and PDAC, based on the Boruta algorithm. (B) Flowchart depicting the experimental design for flow cytometry. PBMCs were isolated from a study cohort consisting of 43 HCs, 39 patients with CP, 45 patients with PDAC and 71 patients with AIP. Flow cytometry analysis was performed on this cohort to analyse the frequency of 10 cell subsets (left) in peripheral blood. (C) Representative flow cytometry plots and box plots showing the proportional variations in IgG4‐high switched memory B cells, Tfh cells and intermediate monocytes among HCs, patients with CP, patients with PDAC and patients with AIP. Statistical differences in these proportions were determined by one‐way ANOVA followed by Dunnett's multiple comparisons test (**p < .01,****p < .0001).

Because the proportions of several cell subsets differed among the three study groups (AIP, PDAC and HCs), we applied the Boruta algorithm on the scRNA‐seq data for feature selection (Figure 5A). This algorithm ranks the proportions of cell subsets according to their importance for disease discrimination. Proportions of B, T, NK and myeloid cell subsets (31 features in total) were included in two separate Boruta models (Figure 5A). Ridge plots were used to visualise the importance of specific cell subsets and the results of feature classification. The proportions of CD4^+^ Tfh cells, IgG4high‐switched memory B cells and intermediate monocytes were identified as common important features in both models.

Next, we developed a 14‐colour flow cytometry gating strategy to identify the above cell subsets and evaluate their potential utility in distinguishing between diseases (Figure S6A). We additionally enrolled a total of 71 treatment‐naïve patients with AIP, 43 HCs and 45 PDAC. A total of 39 patients with CP were included as a disease control group representing common pancreatic disorders (Figure 5B).

Comparisons of peripheral blood immune cell profiles between AIP patients and HCs, CP patients or PDAC patients are shown in Figures 5C and S6B. Relative to HCs, AIP patients exhibited significantly higher proportions of IgG4high‐switched memory B cells, CD3^+^ T cells, CD4^+^ T helper cells, CD4^+^ Tfh cells and intermediate monocytes, alongside a significantly lower proportion of CD19^+^ B cells (Figures 5C and S6B). When comparing AIP patients with CP or PDAC patients, only three subsets: IgG4high‐switched memory B cells, CD4^+^ Tfh cells and intermediate monocytes, showed consistently higher proportions in the AIP group.

### 3.7 Disease discrimination model development, nomogram construction and prognostic value of key cell subsets in AIP

Using flow cytometry data of IgG4high‐switched memory B cells, CD4^+^ Tfh cells and intermediate monocytes, we performed receiver operating characteristic (ROC) analysis. Among these subsets, IgG4high‐switched memory B showed the best performance for distinguishing AIP from HCs, CP and PDAC (Figure S7A). Tfh cells and intermediate monocytes showed comparable performance in distinguishing AIP from HCs and PDAC, whereas Tfh cells appeared to perform better than intermediate monocytes in distinguishing AIP from CP (Figure S7A). ROC analysis was also conducted for well‐established AIP biomarkers.^30^ Plasmablasts showed poor discriminatory ability between AIP and any other group (Figure S7B), whereas serum IgG4 exhibited strong diagnostic performance (Figure S7C).

**FIGURE 6:**
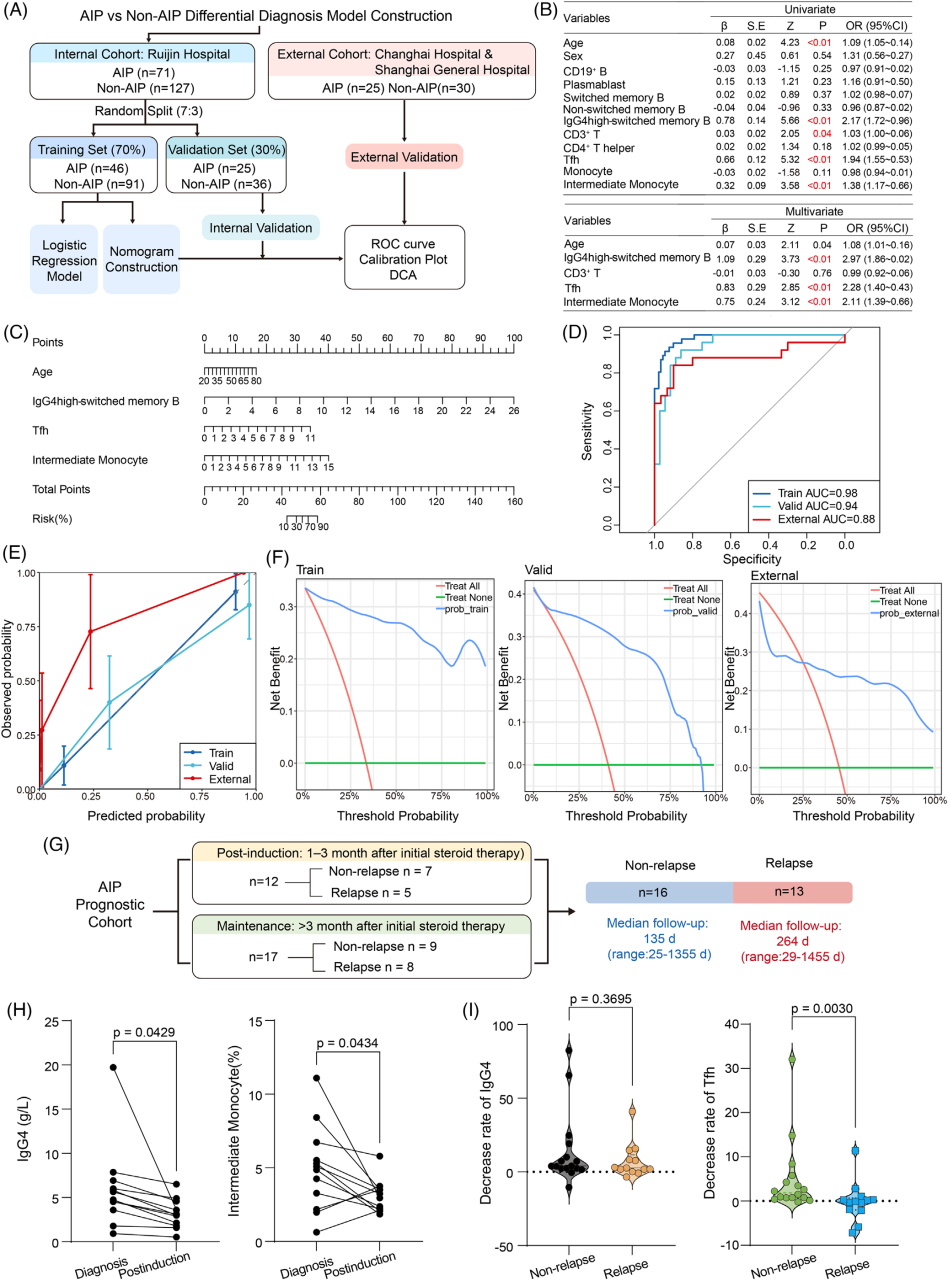
Development and validation of an AIP diagnostic model and the prognostic value of Tfh cells and intermediate monocytes in AIP patients. (A) Study flowchart for model development and validation. (B) Univariate and multi‐variable logistic regression analyses for candidate predictors of AIP. Age, IgG4 high‐switched memory B cells, Tfh cells and intermediate monocytes were retained in the final multi‐variable model. (C) Nomogram for individualised estimation of the probability of AIP. The nomogram was constructed based on age, IgG4 high‐switched memory B cells, Tfh cells and intermediate monocytes, with total points corresponding to the predicted risk of AIP. (D) ROC curves of the model in the training, internal validation and external validation cohorts. (E) Calibration plots of the diagnostic model in the training, internal validation and external validation cohorts. (F) DCA of the diagnostic model in the training, internal validation and external validation cohorts. (G) Flowchart of the AIP prognostic cohort and relapse outcomes after steroid therapy. (H) Before–after plots illustrating the changes in the level of IgG4 and proportion of intermediate monocytes in each patient (right). Statistical differences were determined by paired *t*‐test. (I) Violin plot showing the decrease rate of IgG4 and proportion of Tfh cells in the non‐relapse and relapse groups of AIP patients. (Left) Statistical differences were determined by unpaired *t*‐test.

We next sought to develop a diagnostic model integrating multiple immune cell subsets to distinguish AIP from non‐AIP conditions (Figure 6A). Univariable and multi‐variable logistic regression analyses showed that age, IgG4high‐switched memory B cells, Tfh cells and intermediate monocytes remained significant variables in the final model (Figure 6B), with no substantial multi‐collinearity detected (Figure S7D). These four variables were incorporated into a nomogram (Figure 6C). The model achieved area under the curves (AUCs) of .98 in the training set, .94 in the internal validation set and .88 in the external validation set (Figure 6D). Calibration appeared better in the internal cohorts than in the external cohort (Figure 6E), whereas decision curve analysis (DCA) suggested potential clinical utility across a relevant range of threshold probabilities (Figure 6F).

We further compared serum IgG4 alone, the four‐variable model and a combined model incorporating both. In the external validation cohort, the four‐variable and combined models showed numerically higher AUCs than serum IgG4 alone, with the combined model showing the highest AUC (Figure S7E). However, DeLong's test did not show a statistically significant difference, which may in part reflect the limited sample size of the validation cohort (Figure S7E and Tables S12 and S13). Calibration, DCA (Figure S7F,G) and incremental value analyses (Tables S14 and S15) also appeared to favour the combined model, which is consistent with the possibility that integrating immunophenotypic features with serum IgG4 may improve diagnostic performance.

Finally, to explore the potential effect of steroid therapy on these key immune subsets and to assess their possible prognostic relevance in AIP, we analysed paired follow‐up samples from patients with AIP. The prognostic cohort included 29 patients in total, including 13 patients with relapse and 16 without relapse (Figure 6G). The post‐induction subgroup included 12 patients, with median follow‐up time of 25 days (range, 25–119 days), whereas the maintenance subgroup included 17 patients with median follow‐up time of 349.0 days (range, 123.0–1474 days) (Figure 6G). Follow‐up duration did not differ significantly between the relapse and non‐relapse groups (Figure S8A). After steroid induction therapy, serum IgG4 levels and intermediate monocyte proportions were significantly reduced, whereas IgG4high‐switched memory B cells and Tfh cells did not change significantly (Figures 6H and S8B). In the maintenance subgroup, none of the evaluated markers showed a significant difference from baseline (Figure S8C). The decrease rates of serum IgG4, IgG4high‐switched memory B cells and intermediate monocytes did not differ significantly between relapse and non‐relapse groups, whereas the decrease rate of Tfh cells was significantly greater in the non‐relapse group (Figures 6I and S7D). These findings may suggest that quicker reduction in circulating Tfh cells is associated with lower risks of relapse in AIP.

## 4 DISCUSSION

The understanding of alterations in the peripheral immune landscape of patients with AIP remains limited, which poses challenges for both the identification of disease‐associated biomarkers and the optimisation of clinical management strategies.

By integrating scRNA‐seq and flow cytometry, we identified several immune cell subsets that were enriched in the PBMCs of AIP patients. These subsets showed distinct functional states and patterns of cellular crosstalk that may be relevant to disease pathogenesis and may differ from those observed in PDAC. In addition, some of these subsets may represent candidate biomarkers for distinguishing patients with AIP from both healthy individuals and patients with PDAC.

B cells are widely recognised as important contributors of IgG4‐RD and AIP, as reflected by the clinical efficacy of B cell‐targeting therapies such as rituximab (anti‐CD20) and inebilizumab (anti‐CD19) in both induction and maintenance treatment of IgG4‐RD.^31^, ^32^ Our analyses showed a reduction of peripheral B cells which might be related to their differentiation to plasma cells.^33^, ^34^ Notably, within the B cells, several subsets, including plasmablasts, IgD^+^ ABCs and IgG4high‐switched memory B cells, were significantly increased in the PBMCs of AIP patients. In IgG4‐RD, circulating plasmablasts exhibit oligoclonal expansion and secrete pro‐fibrotic molecules.^7^, ^35^ Although plasmablasts have been proposed as a potential biomarker for AIP,^30^ our data suggest that their performance may be limited. ABCs, which have been implicated in multiple autoimmune diseases, were also recently found to be elevated in AIP.^12^, ^36^ However, given their low proportion in PBMCs and their expression of the cell surface marker CD11c,^37^ ABCs may be less suitable as practical biomarkers.

IgG4 class switched memory B cells have been reported to arise in mature GCs under chronic antigenic stimulation and to retain their memory properties and are long lived.^38^ To our knowledge, this is the first study to identify this subset in the PBMCs of AIP patients. The results further suggest that these cells may have the potential to differentiate into plasma cells in AIP pancreas and that they express relatively high levels of CD23. CD23 is typically expressed on naïve B cells and is down‐regulated during differentiation into memory B cells.^37^ However, previous studies have reported relatively high CD23 expression on IgG4^+^ memory B cells.^39^ Importantly, these IgG4high‐switched memory B cells also express high levels of IgE, and elevated IgE levels have been reported in patients with IgG4‐RD.^40^ This raises the possibility that the Fcε receptor (CD23) on IgG4high‐switched memory B cells may enhance their capacity to interact with IgE‐related pathways. Whether this contributes to the regulation of allergic IgE‐mediated responses in IgG4‐RD remains speculative and will require further investigation.

Moreover, our data suggest that IgG4high‐switched memory B cells may have antigen‐presenting features relevant to both CD4^+^ Tfh cells and CD8^+^ KIR^+^ cytotoxic T cells. IgG4high‐switched memory B cells showed high *AICDA* expression, which may be consistent with a somatically mutated B cell state and a potentially enhanced capacity for self‐antigen capture. Notably, CD4^+^ T cells and CD8^+^ T cells have been shown to induce cell apoptosis in IgG4‐RD, contributing to fibrosis and organ dysfunction.^8^, ^9^ Therefore, it is possible that IgG4high‐switched memory B cells may participate in antigen presentation, thereby contributing to local inflammation or tissue injury. Our study also identified several BCR clonotypes in IgG4high‐switched memory B cells that were shared with plasma cells within the AIP pancreas. However, whether these antibodies are autoreactive remains unknown and should be addressed in the future.

Consistent with previous scRNA‐seq studies, we observed an increase in Tfh cells in the PBMCs of AIP patients. In our analysis, Tfh cells in AIP more closely resembled CXCR5^low^PD1^high^Tph and characterised by high expression of CXCR3 and effector molecules such as CTLA4, GATA3, LAG3 and granzyme A. Masuo et al. recently reported that stem cell‐like Tph cells (S‐T_PH_ cells) can differentiate into effector‐like subset (E‐T_PH_ cells) in patients with rheumatoid arthritis.^29^ Notably, E‐T_PH_ cells appear to resemble the Tph cells characterised in our study. This similarity raises an interesting question that in the pancreatic tissues of AIP patients, if there a comparable S‐TPH cell subset exist and could proliferate into E‐TPH cells in PBMCs, thereby facilitating the dissemination of inflammation to multiple organs. Intriguingly, Tfh cells from patients with AIP showed a more prominent exhaustion‐like transcriptional signature than those from PDAC patients. This observation may reflect distinct immune dysregulation in AIP and could be relevant to the altered cancer susceptibility reported in these patients, although this potential link requires further functional and clinical validation.^13^, ^14^

Myeloid cells also play important roles in the pathogenesis of AIP. Recent studies have reported that *MERTK* ^+^ macrophages infiltrate affected tissues in IgG4‐RD, where they likely mediate efferocytosis of apoptotic cells and secrete pro‐fibrotic cytokines.^10^ Notably, circulating *MERTK* ^+^ monocytes do not appear to be elevated in PBMCs of IgG4‐RD patients, suggesting that *MERTK* ^+^ macrophages may be activated mainly at disease sites rather than in the periphery.^10^ In our analysis, intermediate monocytes were increased in patients with AIP and showed antigen‐presentation‐related features, a potential capacity to differentiate into multiple macrophage states in the AIP pancreas, and up‐regulated *TREM2* expression. We have previously reported enrichment of *TREM2* ^+^ macrophages in the pancreas of AIP patients.^12^ Given recent evidence that *TREM2* may function as an important signalling mediator in macrophages and may be associated with protective effects against fibrosis,^41^ our finding that intermediate monocytes up‐regulate *TREM2* is consistent with the possibility that these monocytes may serve as progenitors of such macrophages. However, this interpretation remains speculative and will require direct experimental validation.

Our study observed several potentially meaningful links between peripheral blood and pancreatic immune changes, such as pancreatic tissue showed marked plasma‐cell enrichment despite only a modest increase in circulating plasmablast/plasma‐cell proportions, and circulating ABCs differed phenotypically from their tissue counterparts. However, peripheral blood did not fully capture several tissue‐specific immune changes, including the diverse differentiation states of pancreatic B cells and the heterogeneity of tissue‐resident macrophages. Although Tfh cells were altered in both blood and pancreas, we should cautiously interpret this finding, as circulating immune changes may also reflect immune activity in other organs involved in IgG4‐RD. Thus, the blood–pancreas relationships described here are best considered inferential rather than directly equivalent.

Several limitations should be acknowledged. First, due to the low proportion of IgG4high‐switched memory B cells, we were unable to isolate sufficient numbers of this pure subset to perform direct siRNA transfection and functional assays. Future studies with larger sample availability will be needed to enable more direct functional experiments on this subset. Second, age may have influenced immune cell distributions because the cohort was not fully age‐matched, despite adjustment in the multi‐variable model. In addition, all validation centres were located within the Shanghai region, and calibration in the external cohort was less optimal than that in the internal cohorts, which may limit broader generalisability. Finally, the prognostic analysis was based on a relatively small cohort with limited follow‐up compared with recent relapse studies in AIP,^26^, ^42^, ^43^ partly because serial whole‐blood sampling at multiple time points is difficult in longitudinal studies. Therefore, the relapse‐related findings should be interpreted as preliminary and warrant further validation.

In conclusion, through scRNA‐seq, we identified several immune cells that were enriched in the PBMCs of AIP patients, characterised their phenotypic features and potential pathogenic relevance. Multi‐colour flow cytometry experiments supported their possible utility in diagnosis, particularly in differential diagnosis and suggested a potential association with patient prognosis. Our findings deepen the understanding of AIP pathogenesis and may provide a basis for improving the clinical management of this disease.

## AUTHOR CONTRIBUTIONS

Chunhua Zhou, Duowu Zou and Minmin Zhang conceived and designed the research and supervised the studies. Chenxiao Liu and Tianyi Che conducted analyses. Jiaxin Wang and Youqiong Ye guided supervised analyses. Tianyi Che, Xiaonan Shen, Xiangyi He, Tingting Gong, Ling Zhang, Junjie Fan, Yue Zeng and Wenbin Zou conducted the clinical cohort and collected samples. Chenxiao Liu, Qidi Yang, Yiwen Tu and Zonghao Liu performed the experiments. Chenxiao Liu and Airu Liu wrote the manuscript. Zhengji Song, Yao Zhang, Chunhua Zhou and Duowu Zou revised the manuscript. All authors read and approved the final manuscript.

## CONFLICT OF INTEREST STATEMENT

The authors declare no conflicts of interest.

## ETHICS STATEMENT

This study was approved by local medical ethics boards from Ruijin Hospital Affiliated to Shanghai Jiao Tong University (2021‐No.301) and conducted in compliance with the principles of the Declaration of Helsinki.

## CONSENT

All included patients provided informed written consent and patient assent. Patients or the public were not involved in the design, conduct, reporting or dissemination plans of this research.

## CODE AVAILABILITY STATEMENT

No new algorithms were developed for this manuscript. All code generated for analysis is available from the authors upon request.

## Supporting information



Supporting Information

Supporting Information

Supporting Information

Supporting Information

Supporting Information

Supporting Information

Supporting Information

Supporting Information

Supporting Information

## Data Availability

The raw sequence data of all scRNA‐seq and BCR‐seq for PBMCs of AIP patients in this paper have been deposited in the GSA (Genomics, Proteomics & Bioinformatics 2025)^44^ in National Genomics Data Center (Nucleic Acids Res 2025),^45^ China National Center for Bioinformation/Beijing Institute of Genomics, Chinese Academy of Sciences (GSA‐Human: HRA014901) that are publicly accessible at https://ngdc.cncb.ac.cn/gsa‐human.
