# Supplementary figures and images for: Single‐cell RNA sequencing and high‐dimensional flow cytometry reveal distinct peripheral immune landscapes of type 1 autoimmune pancreatitis and pancreatic ductal adenocarcinoma
Source: Clin Transl Med. 2026 Apr 21;16(4):e70680. doi: 10.1002/ctm2.70680 (PMC13097352; doi:10.1002/ctm2.70680)

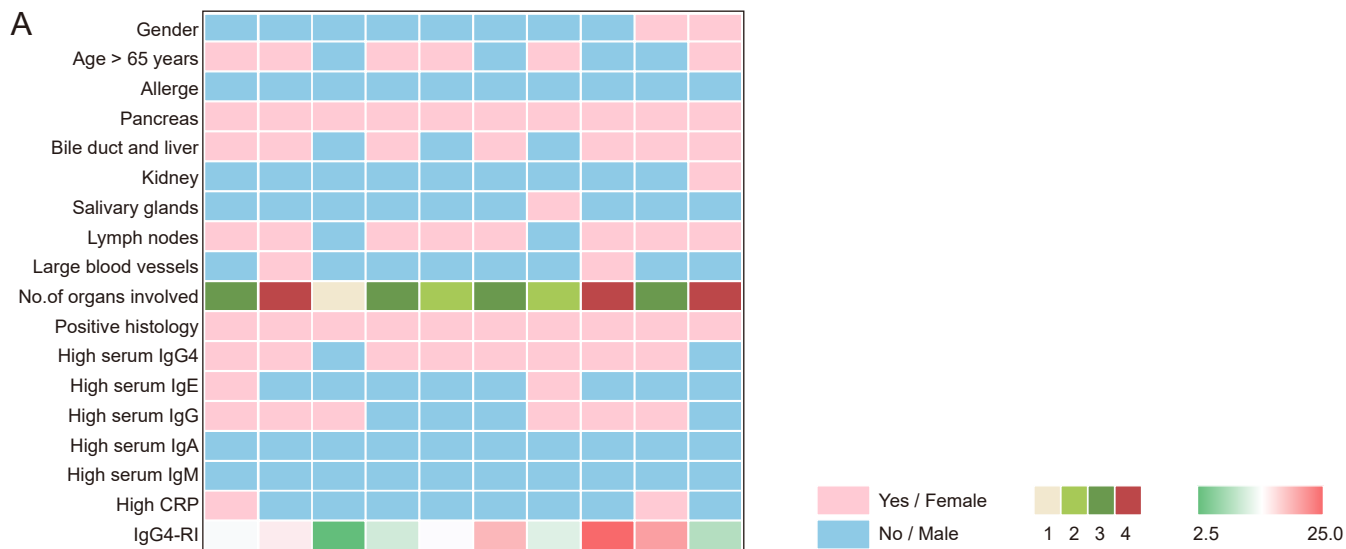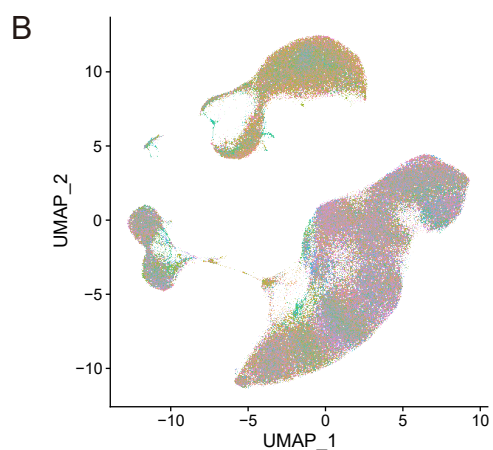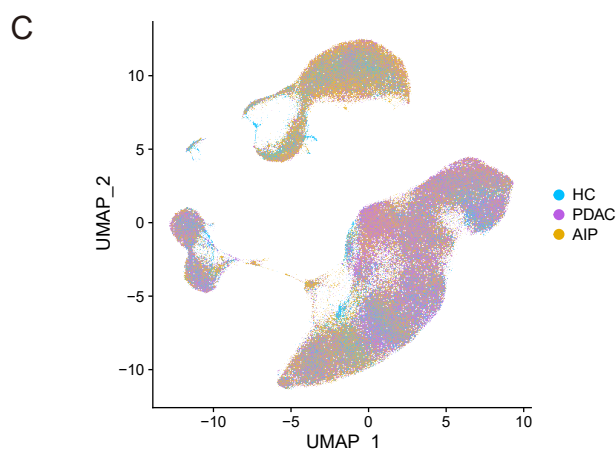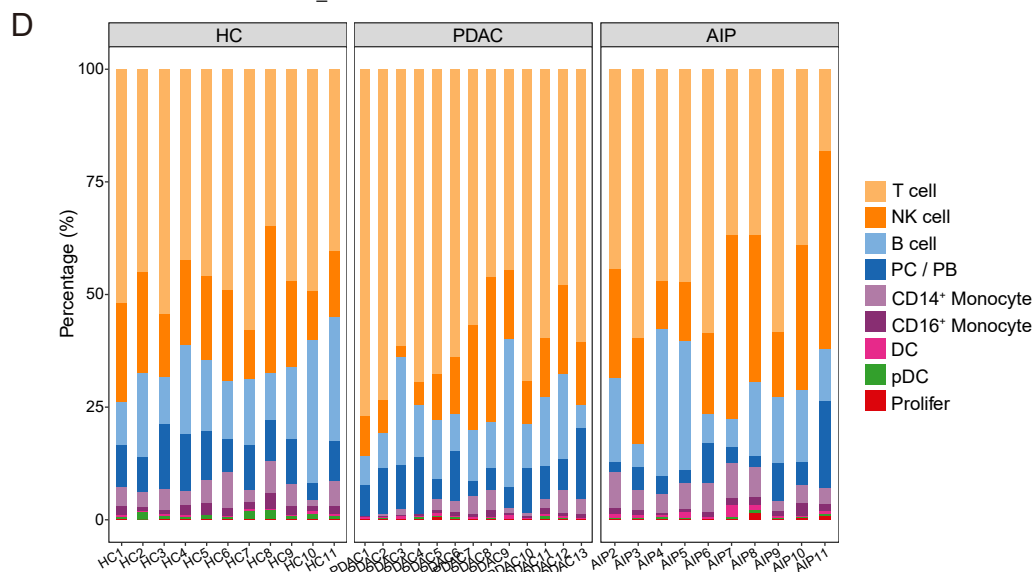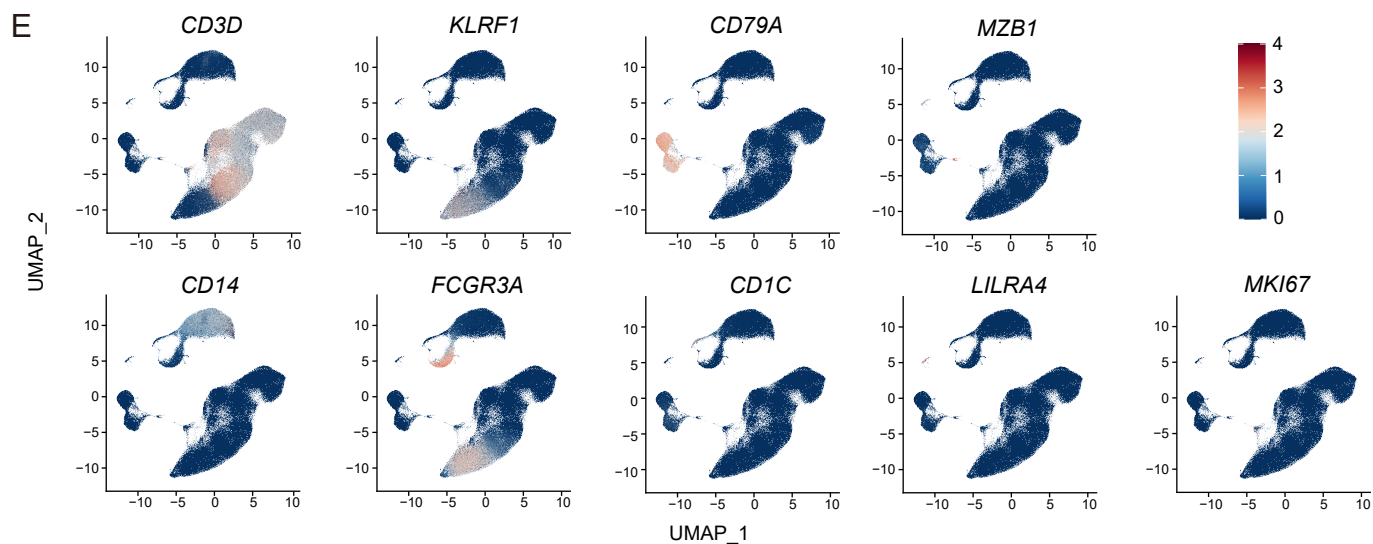

Supplement: Supplementary file 1 — Supporting Information [file CTM2-16-e70680-s005.pdf]

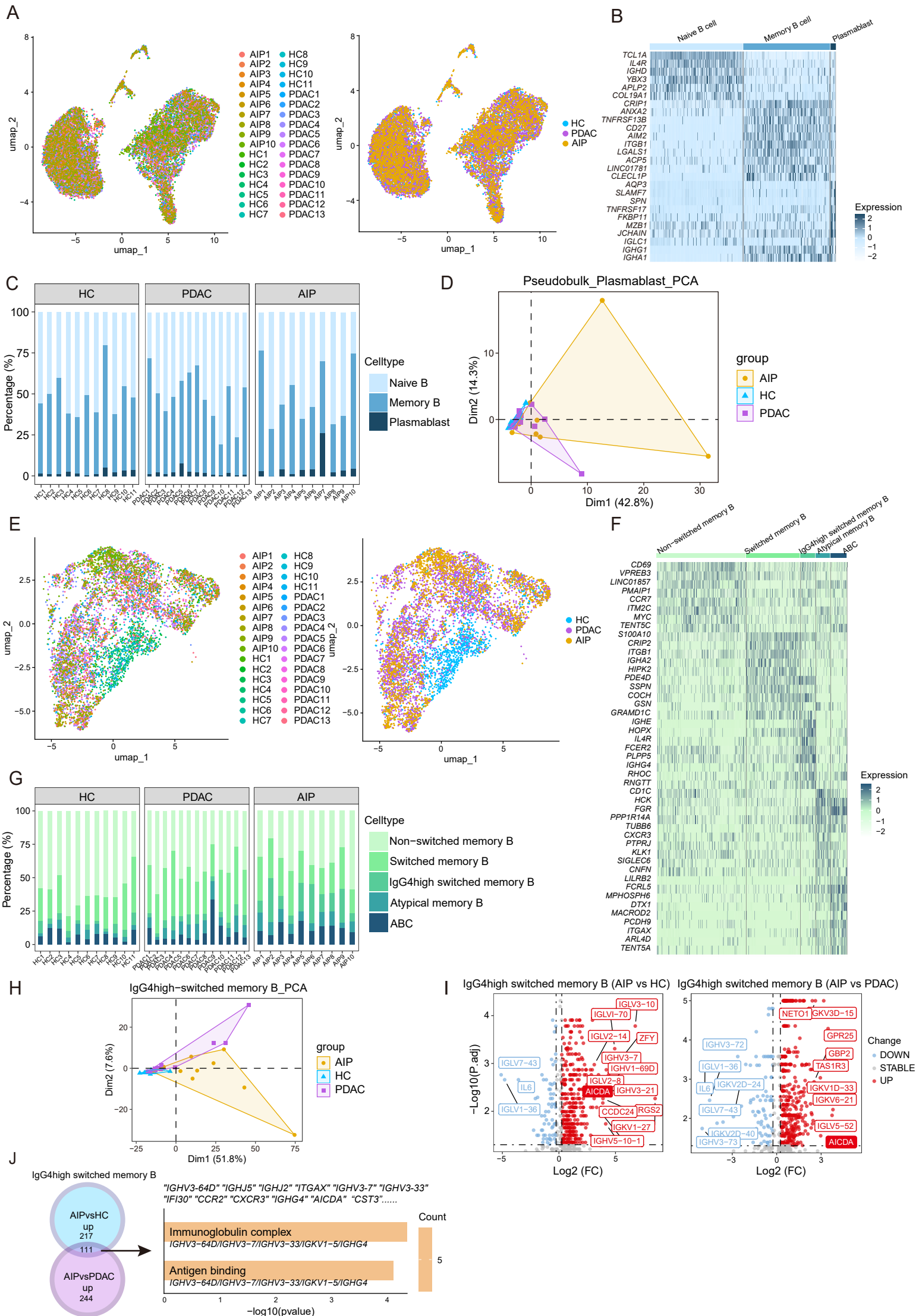

Supplement: Supplementary file 2 — Supporting Information [file CTM2-16-e70680-s009.pdf]

A

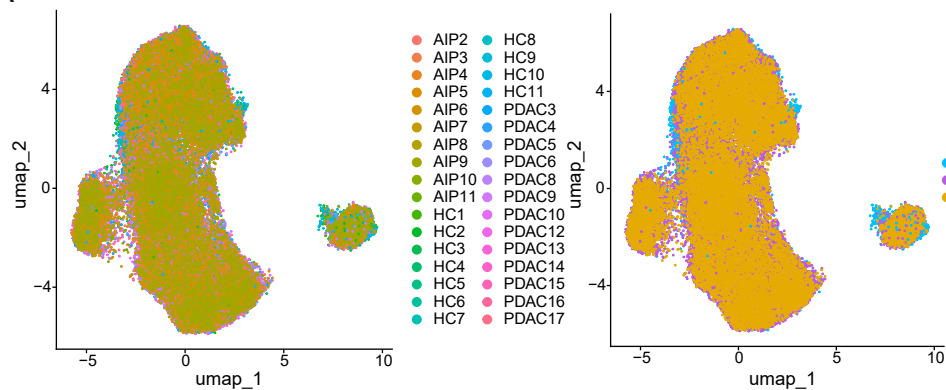

B

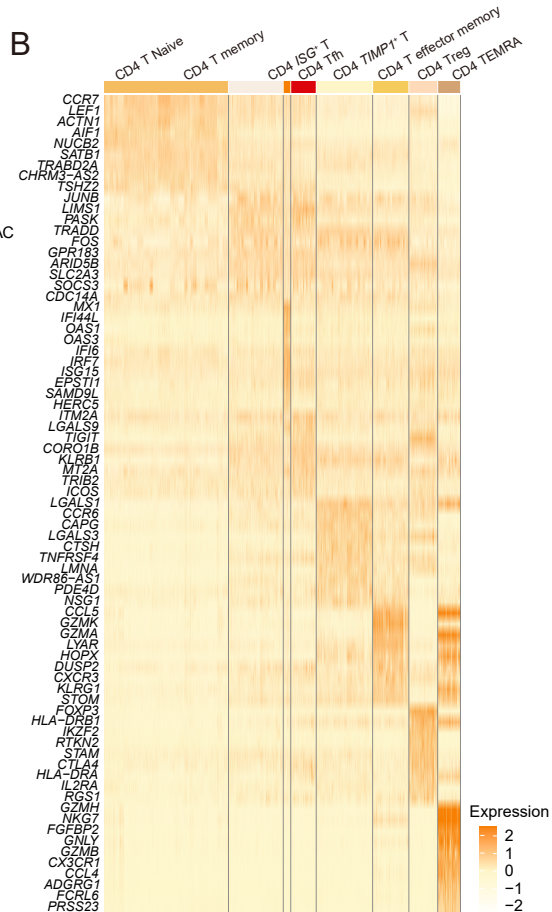

C

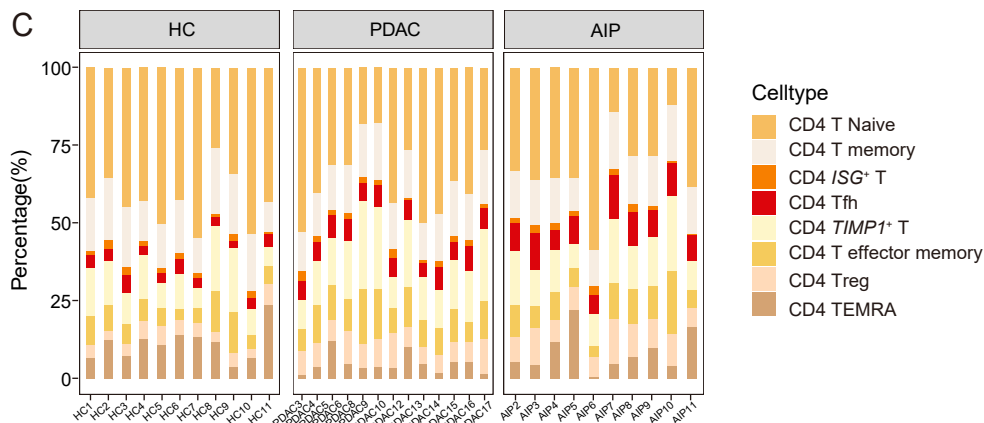

D

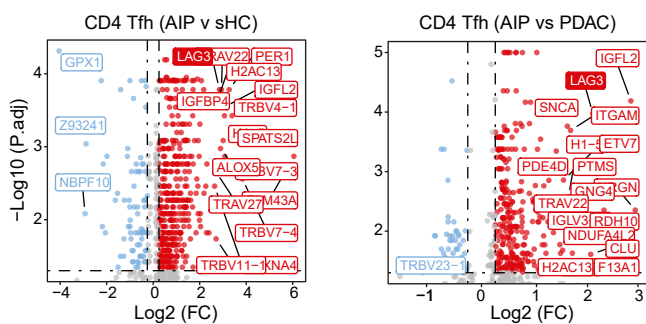

E

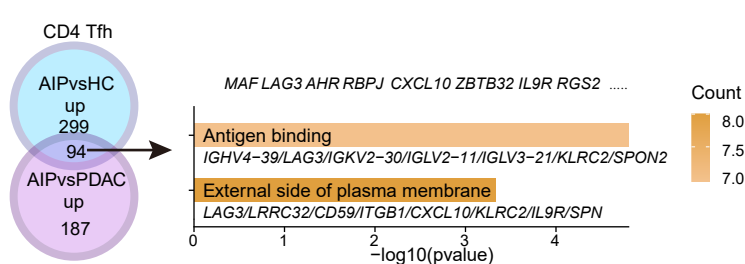

F

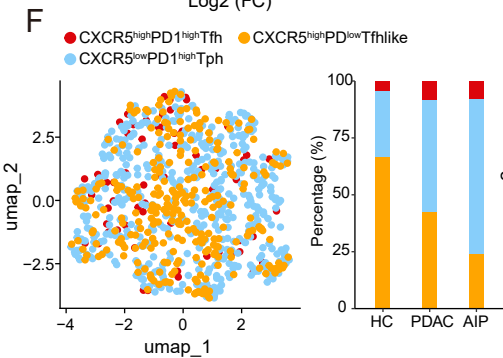

G

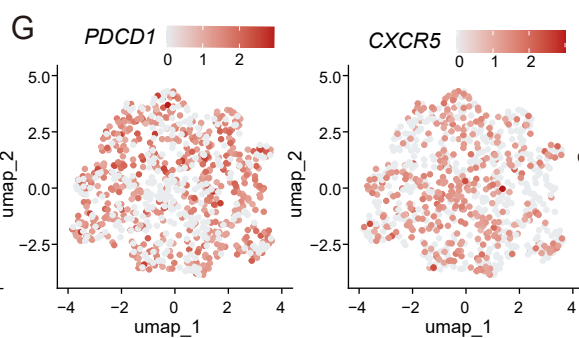

H

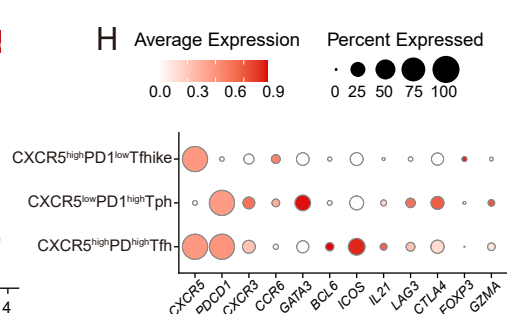

I

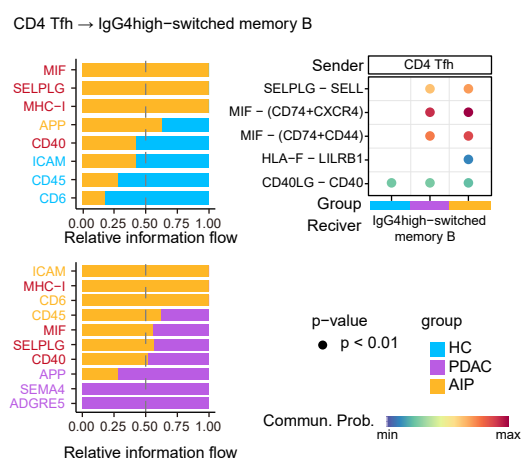

J

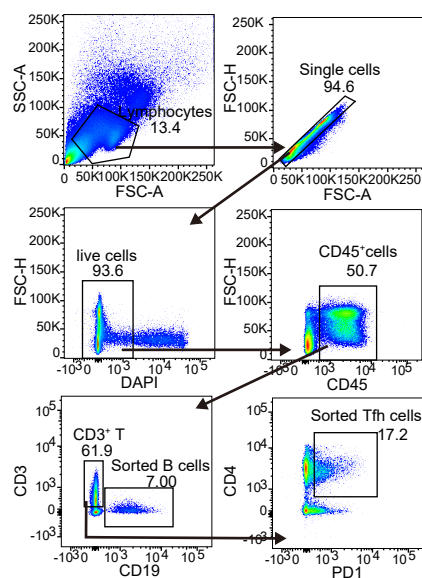

K

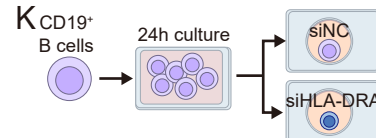

L

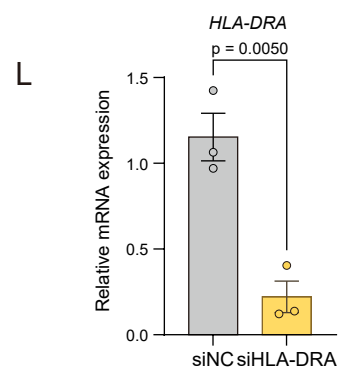

Supplement: Supplementary file 3 — Supporting Information [file CTM2-16-e70680-s001.pdf]

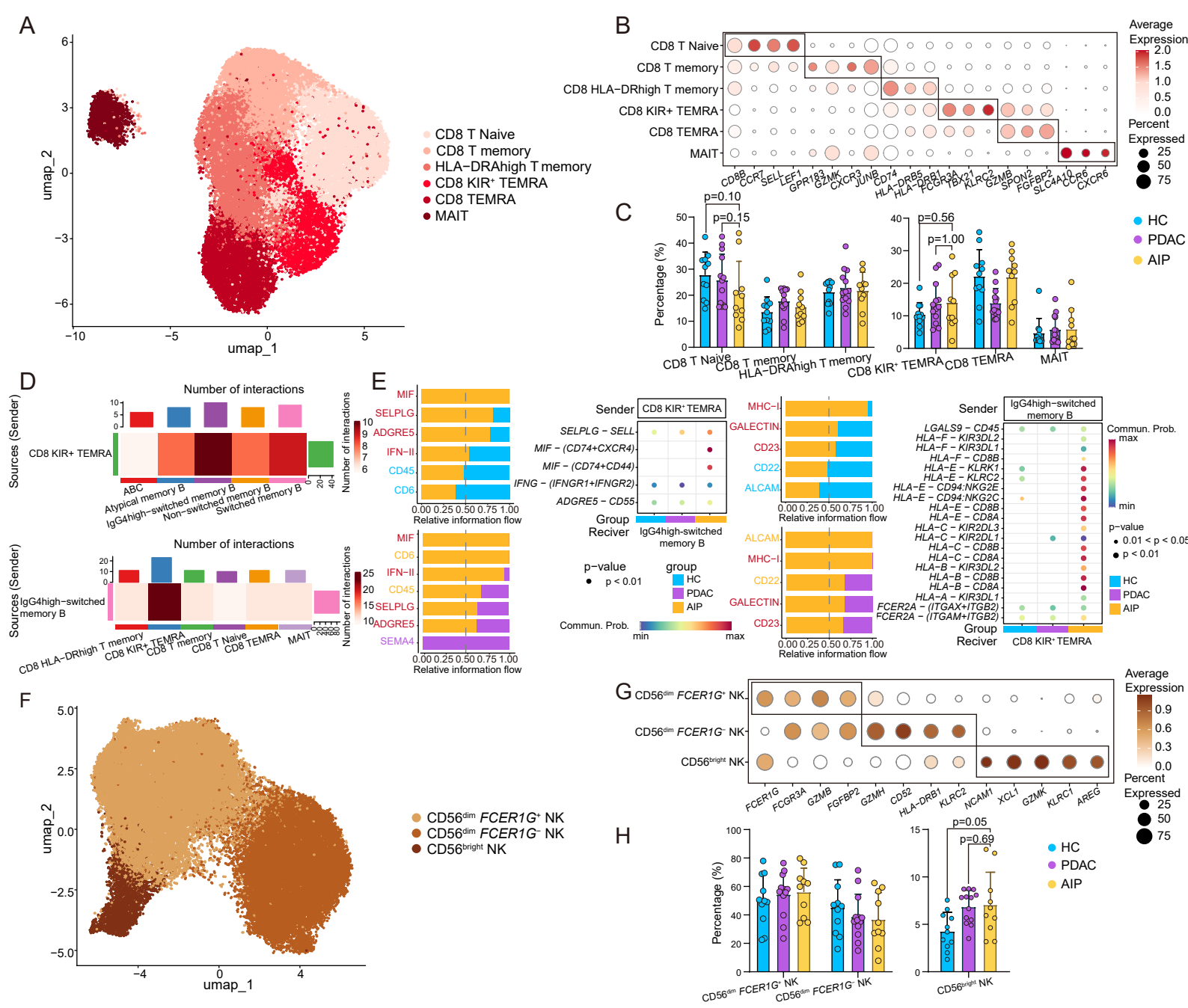

Supplement: Supplementary file 4 — Supporting Information [file CTM2-16-e70680-s003.pdf]

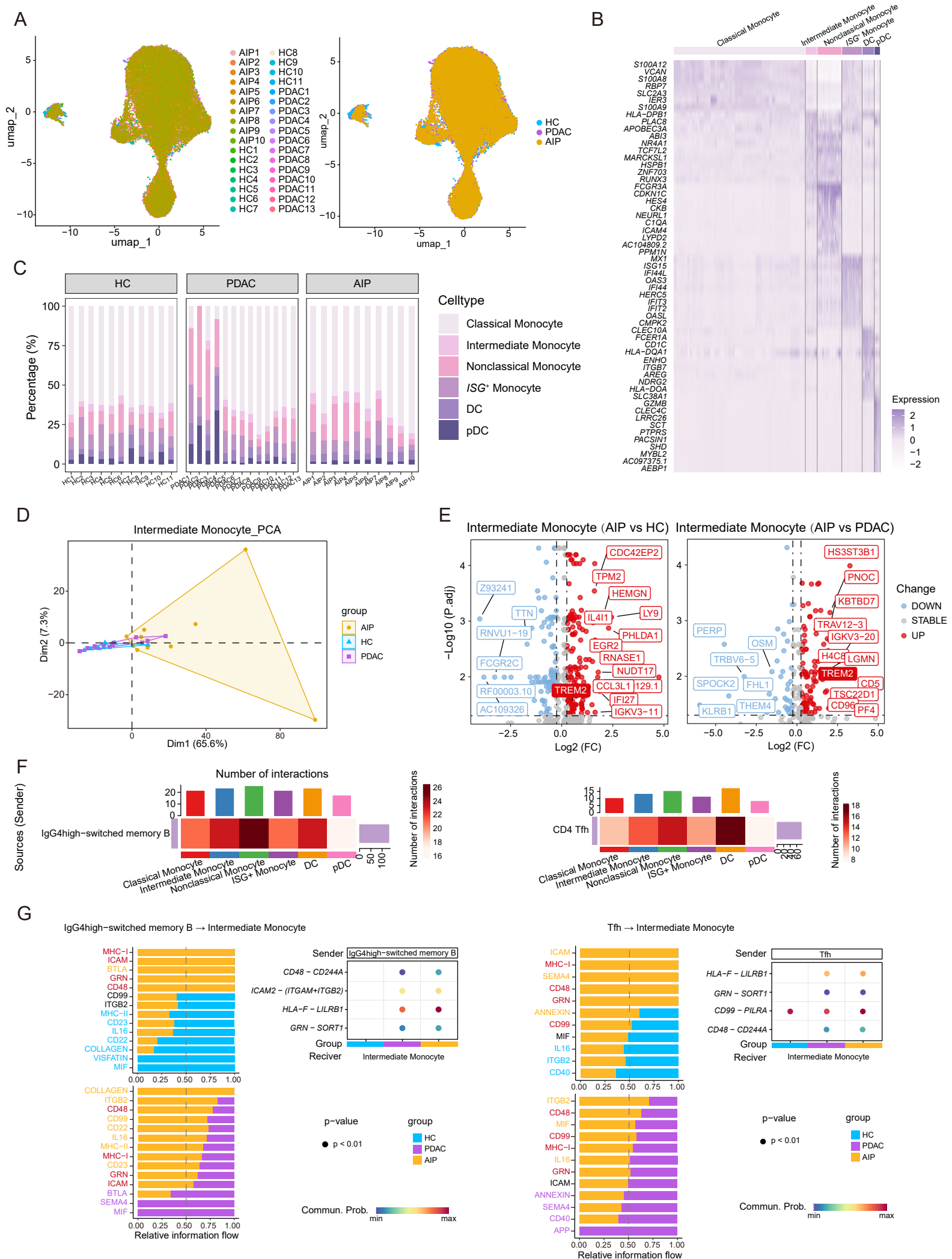

Supplement: Supplementary file 5 — Supporting Information [file CTM2-16-e70680-s006.pdf]

A

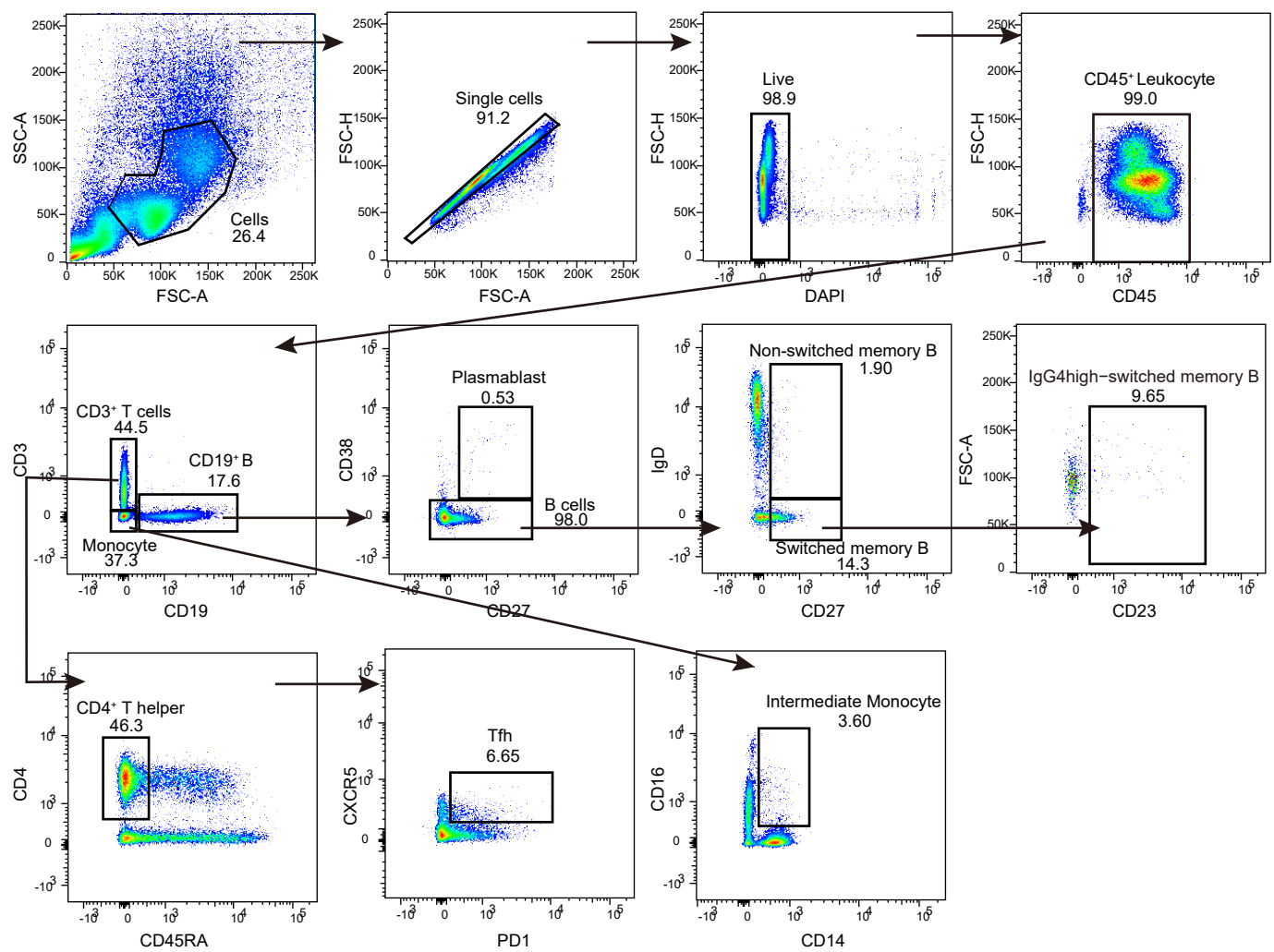

B

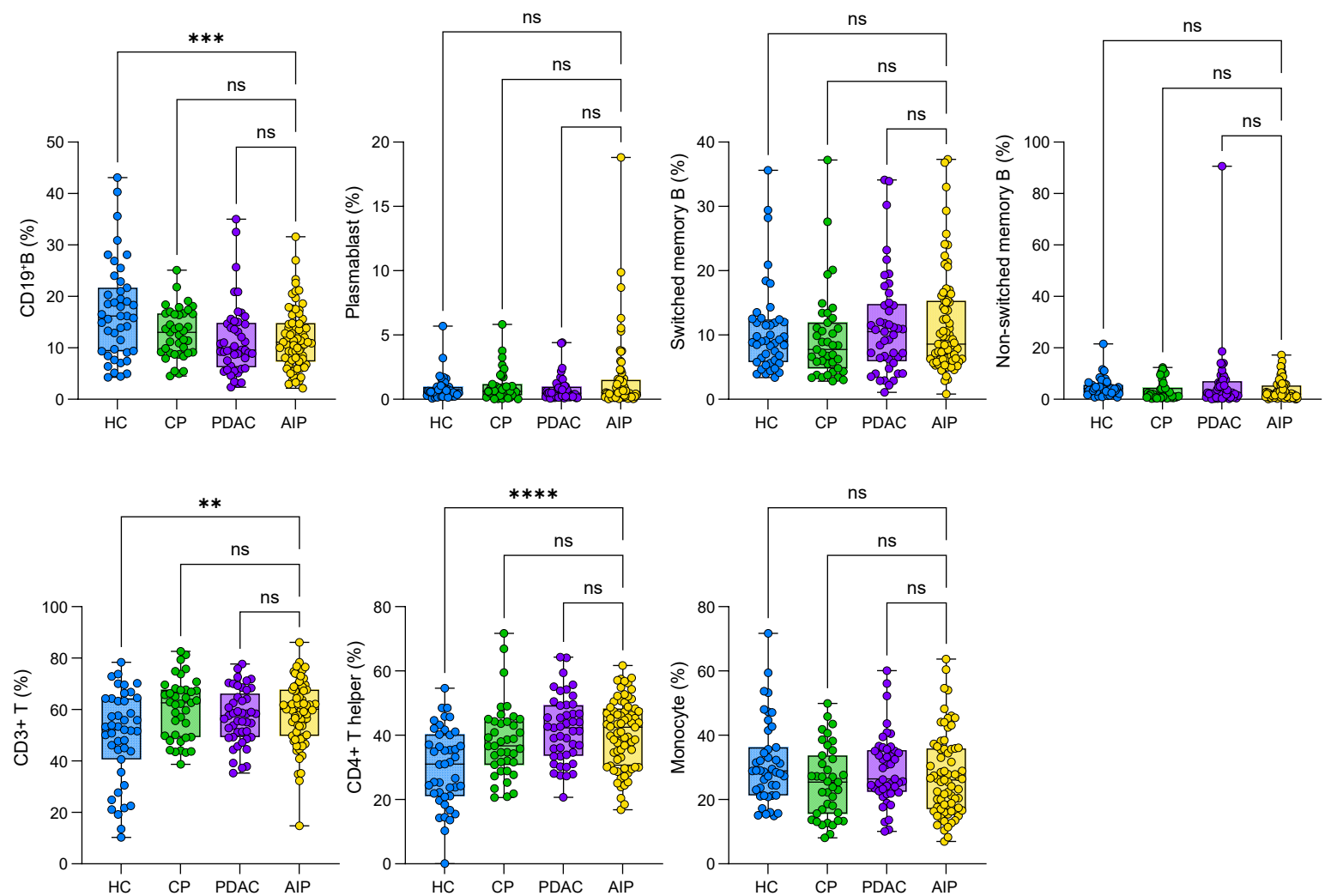

Supplement: Supplementary file 6 — Supporting Information [file CTM2-16-e70680-s007.pdf]

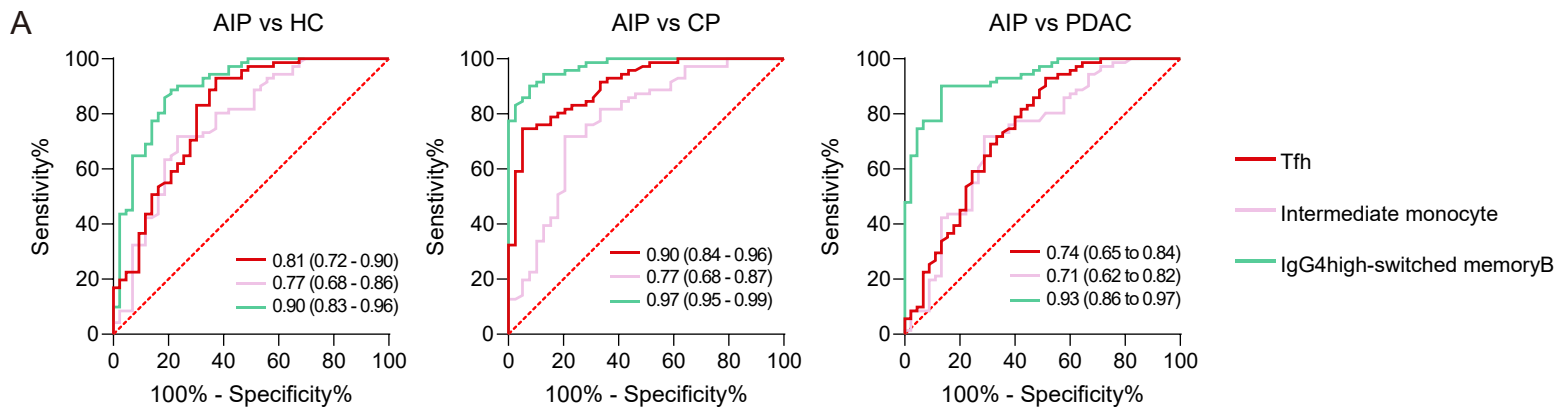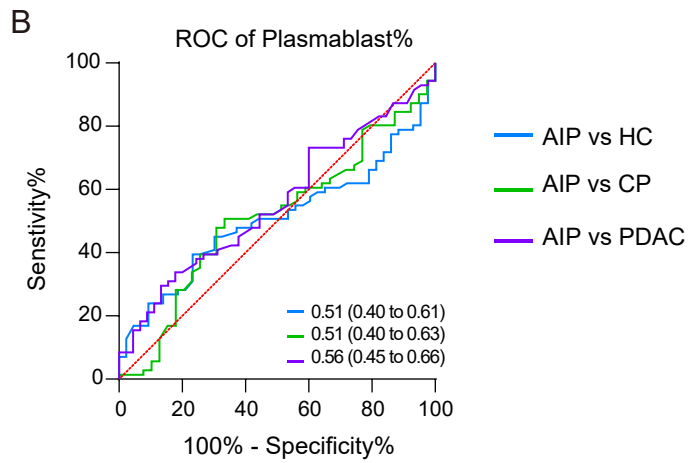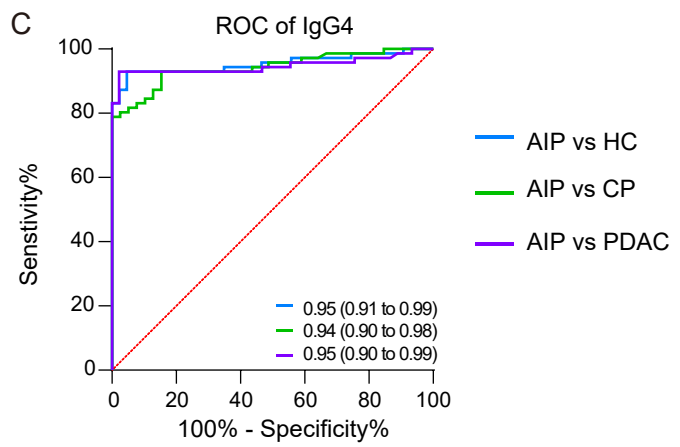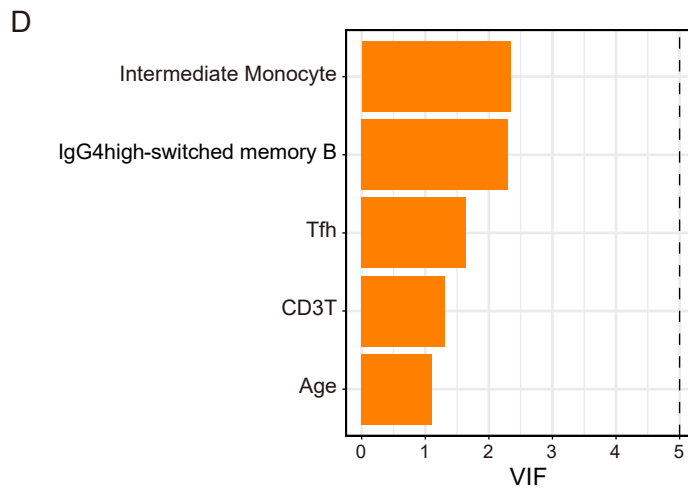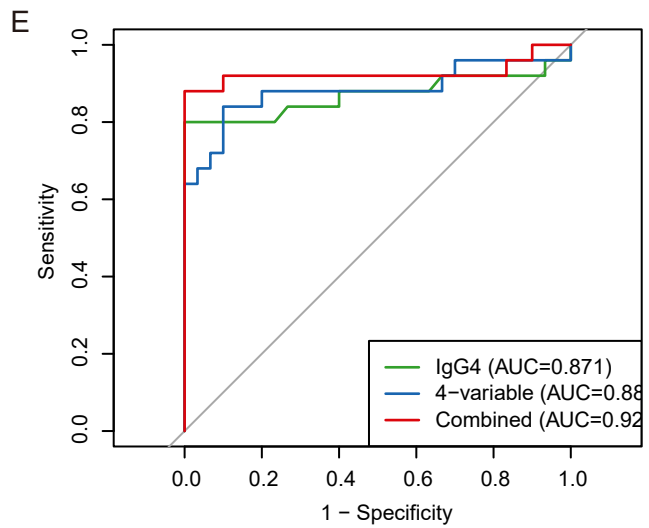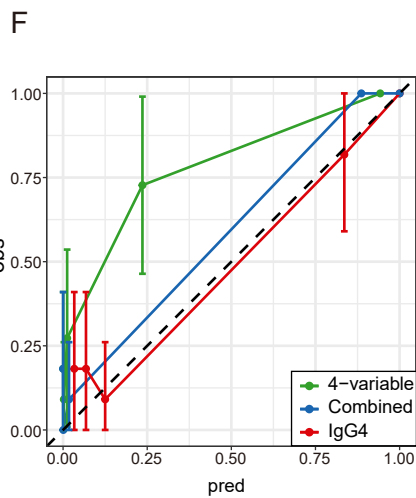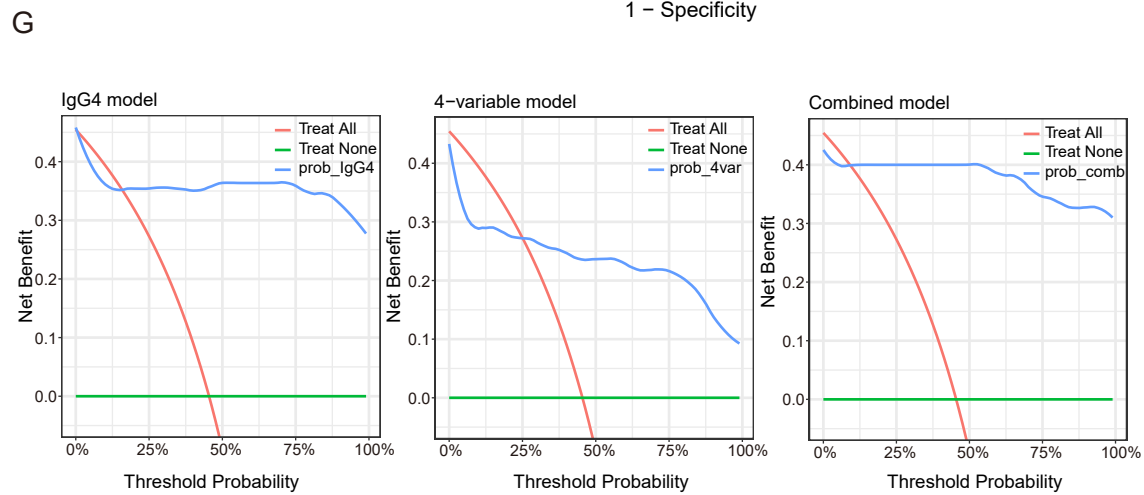

Supplement: Supplementary file 7 — Supporting Information [file CTM2-16-e70680-s002.pdf]

A

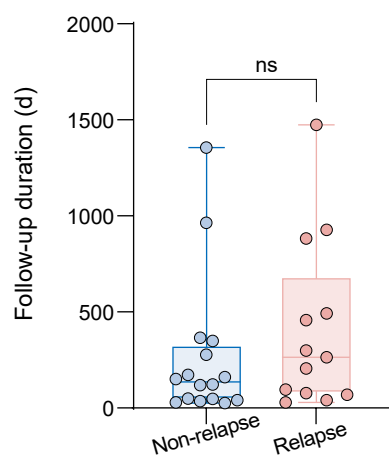

B

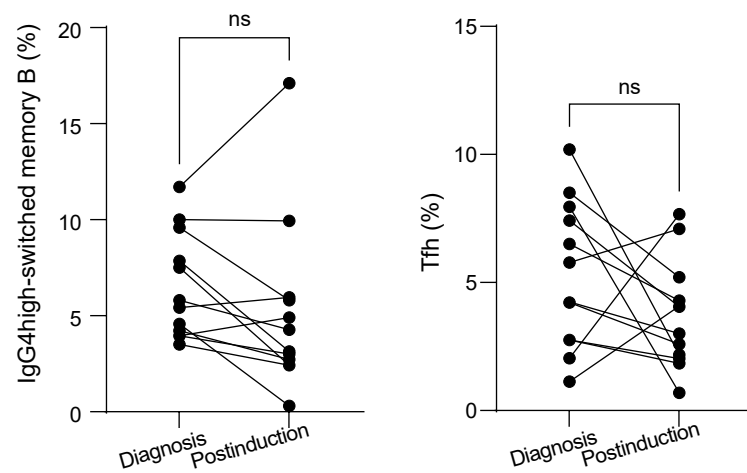

C

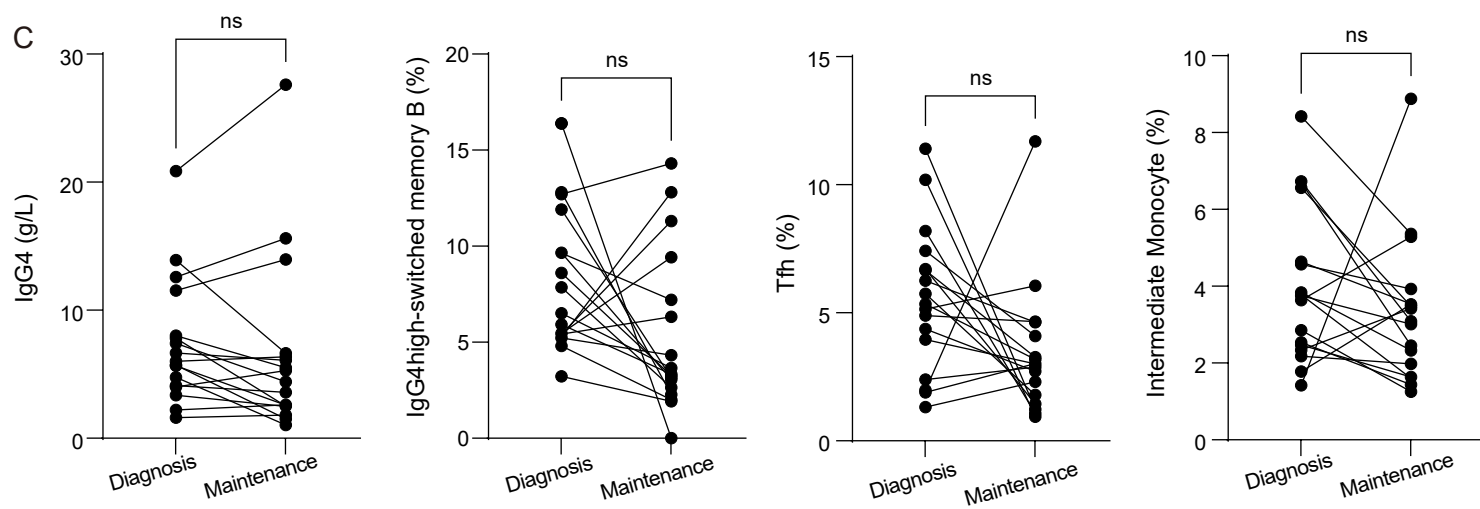

D

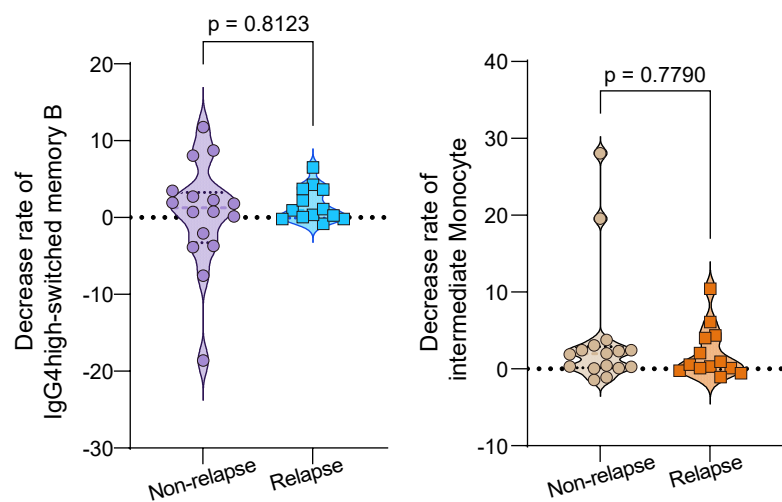

Supplement: Supplementary file 8 — Supporting Information [file CTM2-16-e70680-s004.pdf]
