# Supplementary material for: Single‐cell RNA sequencing and high‐dimensional flow cytometry reveal distinct peripheral immune landscapes of type 1 autoimmune pancreatitis and pancreatic ductal adenocarcinoma
Source: Clin Transl Med. 2026 Apr 21;16(4):e70680. doi: 10.1002/ctm2.70680 (PMC13097352; doi:10.1002/ctm2.70680)
Supplement: Supplementary file 9 — Supporting Information [file CTM2-16-e70680-s008.docx]

**SUPPLEMENTARY MATERIAL**

**Flow Cytometry**

Frozen PBMCs were thawed and resuspended in PBS containing 2% FBS and 2 mM EDTA (FACS buffer). To reduce nonspecific protein binding, cells were incubated with human Fc Block (1:100) at 4℃ for 10 min. Antibodies for immune cells were diluted in FACS buffer with appropriate concentrations. For surface marker staining, cells were stained for 30 min at 4 °C with the following antibodies: Anti-CD45 (BD Horizon, 563792, 1:200), anti-CD19 (BioLegend, 363024, 1:200), anti-CD3 (BD Horizon, 300463, 1:200), anti-CD38 (BioLegend, 303524, 1:200), anti-CD27 (BioLegend, 302815, 1:200), anti-IgD (BioLegend, 348240, 1:100), anti-CD23 (BioLegend, 338507, 1:200), anti-CD4 (BioLegend, 317434, 1:200), anti-CD45RA (BioLegend, 304125, 1:200), anti-CXCR5 (BioLegend, 356913, 1:100), anti-PD-1 (BioLegend, 329910, 1:200), anti-CD14 (BioLegend, 301841, 1:200), anti-CD16 (BioLegend, 302041, 1:200), and anti-HLA-DR (BD Horizon, 566480, 1:200). Dead cells were identified by DAPI staining. After staining, cells were washed twice. Flow cytometry was performed on a BD Symphony (BD Biosciences). Data were acquired using BD FACSDiva software v8.0.2, then analyzed with FlowJo v10.8.1 (Tree Star Inc).

The proportions of lymphocyte subsets were recorded as follows: CD19^+^ B cells, CD3^+^ T cells and CD19^-^CD3^-^ monocytes proportions were referred to the percentage of them in CD45^+^ lymphocytes; plasmablast proportions were referred to the percentages of them in CD19^+^ B cells; IgD^-^ switched memory B cells and IgD^+^ non-switched memory B cells were referred to the percentages of them in CD27^+^CD38^-^ memory B cells; CD23^+^ IgG4high-switched memory B cells were referred to the percentages of them in IgD^-^ switched memory B; CD4^+^CD45RA^-^ T helper cells proportions were referred to the percentage of them in CD3^+^ T cells; PD1^+^CXCR5^+^ Tfh cells proportions were referred to the percentage of them in CD4^+^CD45RA^-^ T helper cells; CD14^+^CD16^+^ Monocytes were referred to the proportions of them in CD19^-^CD3^-^ monocytes.

**Reverse Transcription Quantitative Polymerase Chain Reaction (RT-qPCR)**

Total RNA was extracted using TRIzol reagent (Invitrogen). Complementary DNA (cDNA) was synthesized using the SuperScript III cDNA Synthesis Kit (Invitrogen). Quantitative PCR was performed using SYBR Green on the LightCycler PRO system (Roche) (primers in Table 2). Relative gene expression was calculated using the 2^^−ΔΔCt^ method, normalized to GAPDH.

**Diagnostic model construction and validation**

Model performance was evaluated in the training, internal validation, and external validation cohorts. Discrimination was assessed using receiver operating characteristic (ROC) curves and the area under the curve (AUC). Calibration was assessed by grouping individuals according to predicted probabilities and comparing mean predicted and observed event probabilities. Patients were divided into three groups in the training and internal validation cohorts and five groups in the external validation cohort. Clinical utility was evaluated using decision curve analysis (DCA) based on predicted probabilities from the logistic regression model.

For model comparison, three diagnostic models were established in the training set: an IgG4 model based on serum IgG4 alone, a four-variable model, and a combined model including these four variables plus serum IgG4. The predictive performance of these models was evaluated in the external validation cohort. Differences in AUC were compared using the DeLong test. Reclassification and discrimination improvement were further assessed using the net reclassification improvement (NRI) and integrated discrimination improvement (IDI). For NRI analysis, risk categories were predefined using probability cutoffs of 0.3 and 0.7, and 1,000 bootstrap iterations were performed. IDI and its 95% confidence interval were also estimated using 1,000 bootstrap resamples.

**Statistical Analysis**

Continuous variables are presented as mean ± standard deviation (SD) or median (interquartile range [IQR]). Categorical variables, including sex and allergy history, were compared using Fisher’s exact test. In the single-cell analysis of immune cell subset proportions and gene-set scores, pairwise comparisons among groups were performed using the Mann–Whitney U test. To account for multiple testing, Bonferroni correction was applied to control for type I error. The adjusted significance threshold was defined as 0.05 divided by the number of comparisons, and only adjusted P values below this threshold were considered statistically significant. For flow cytometric cell proportion analysis, qPCR, and ELISA data, comparisons among groups were performed using one-way or two-way analysis of variance (ANOVA), followed by Dunnett’s multiple comparisons test, and the resulting adjusted P values were reported. Comparisons of cell subset proportions before and after therapy were performed using a paired t-test, whereas comparisons of decrease rates between the relapse and non-relapse groups were performed using an unpaired t-test. Details of the statistical tests used for individual analyses are provided in the corresponding figure legends. All tests were two-sided, and P < 0.05 was considered statistically significant unless otherwise specified.

**SUPPLEMENTARY FIGURE LEGENDS**

**Fig S1. Clinical Information of AIP Patients Included in scRNA-seq Analysis and Single-Cell Landscape of PBMCs in AIP.**

1. Heatmap showing the clinical information of AIP patients included in the scRNA-seq analysis.
2. UMAP plot showing major cell clusters distribution across PBMCs of 10 AIP patients, 11 HCs and 13 PDAC patients.
3. UMAP plot showing major cell clusters distribution across PBMCs of HCs, AIP patients and PDAC groups.
4. Bar plots showing percentage of major cell clusters across 34 samples.
5. Feature plots showing the expression of canonical markers in major cell clusters.

**Fig S2. Heterogeneity of the B cells in the PBMCs of AIP patients.**

1. UMAP plots showing B cell subsets distribution across PBMCs of 10 AIP patients, 11 HCs and 13 PDAC patients.
2. Heatmap showing top 10 marker genes of indicated B cell subsets.
3. Bar plots showing percentage of B cell subsets across 34 samples.
4. PCA plot showing the results of pseudobulking analysis for plasmablasts, with the three groups in different colors.
5. UMAP plot showing memory B cell subsets distribution across PBMCs of 10 AIP patients, 11 HCs and 13 PDAC patients.
6. Heatmap showing top 10 marker genes of indicated memory B cell subsets.
7. Bar plots showing percentage of memory B cell subsets across 34 samples.
8. PCA plot visualizing the results of pseudobulking analysis for IgG4-high switched memory B cells, with the three groups in different colors.
9. Volcano plot showing the upregulated and downregulated DEGs in the IgG4high-switched memory B cell subset, derived from pseudobulking analysis.
10. Venn plot illustrating the overlap of upregulated DEGs in IgG4high-switched memory B cells. Representative enriched terms from the GO biological process analysis of these common DEGs in plasmablasts are presented in the right.

**Fig S3.** **Heterogeneity of CD4+ T cells in PBMCs from patients with AIP and HLA-DRA knockdown in CD19^+^ B cells.**

1. UMAP plots showing CD4^+^ T cell subsets distribution across PBMCs of 10 AIP patients, 11 HCs and 13 PDAC patients.
2. Heatmap showing top 10 marker genes of indicated CD4^+^ T cell subsets.
3. Bar plots showing percentage of CD4^+^ T cell subsets across 34 samples.
4. Volcano plot showing the upregulated and downregulated DEGs in the CD4+ Tfh cell subset, derived from pseudobulking analysis
5. Venn plot illustrating the overlap of upregulated DEGs in CD4+ Tfh cells. Representative enriched terms from the GO BP analysis of these common DEGs in plasmablasts are shown on the right.
6. (left) UMAP plot showing CXCR5highPD1highTfh cell, CXCR5lowPD1highTph and CXCR5highPD1lowTfhlike cell subsets. (right) Bar plots showing percentage of the three cell subsets across the three disease groups.
7. Feature plots showing the expression of *PDCD1* and *CXCR5* in CD4^+^ Tfh cells.
8. Dot plot displaying the functional genes in the three CD4^+^ Tfh cell subsets.
9. Left: Bar plot showing the information flow from CD4^+^ Tfh cells to IgG4high switched memory B cells. Right: Dot plot illustrating the specific ligand‒receptor communication probabilities.
10. Representative flow cytometry gating strategy for isolating Tfh cells and B cells. Lymphocytes, single cells, live cells, and CD45+ cells were sequentially gated, followed by identification of CD3^+^ T cells, sorted B cells, and sorted Tfh cells.
11. Schematic diagram of the in vitro experiment. CD19^+^ B cells were cultured for 24 h and transfected with negative control siRNA (siNC) or *HLA-DRA* siRNA (si *HLA-DRA*).
12. Relative *HLA-DRA* mRNA expression in CD19^+^ B cells after transfection with siNC or si*HLA-DRA*. Statistical differences were determined by unpaired t-test.

**Fig S4.** **Heterogeneity of the CD8^+^T cells and NK cells in the PBMCs of AIP patients.**

1. UMAP plot showing 6 CD8**^+^** T cell subsets from PBMCs of HCs, PDAC patients and AIP patients in different colors.
2. Dot plot displaying representative markers in each CD8^+^ T-cell subset.
3. Bar plots comparing the percentage of CD8**^+^** T cell subsets. Statistical differences were determined by Mann-Whitney U-test.
4. (top) Heatmap showing the number of interactions from CD8**^+^** KIR^+^ TEMRA (sender) to all memory B cell subsets (receivers). (bottom) Heatmap showing the number of interactions from IgG4high-switched memory B cells (sender) to all CD8**^+^** T cell subsets (receivers).
5. (left) Bar plot showing the information flow from CD8**^+^** KIR^+^ TEMRA to IgG4high switched memory B cells. Right: Dot plot illustrating the specific ligand‒receptor communication probabilities. (right) Bar plot showing the information flow from IgG4high switched memory B cells to CD8**^+^** KIR^+^ TEMRA. Right: Dot plot illustrating the specific ligand‒receptor communication probabilities.
6. UMAP plot showing 3 NK cell subsets from PBMCs of HCs, PDAC patients and AIP patients in different colors.
7. Dot plot displaying representative markers in each NK cell subset.
8. Bar plots comparing the percentage of NK cell subsets. Statistical differences were determined by Mann-Whitney U-test.

**Fig S5. Heterogeneity of the Myeloid cells in the PBMCs of AIP patients.**

1. UMAP plots showing myeloid cell subsets distribution across PBMCs of 10 AIP patients, 11 HCs and 13 PDAC patients.
2. Heatmap showing top 10 marker genes of indicated myeloid cell subsets.
3. Bar plots showing percentage of myeloid cell subsets across 34 samples.
4. PCA plot visualizing the results of pseudobulking analysis for intermediate monocytes, with the three groups in different colors.
5. Volcano plot showing the upregulated and downregulated DEGs in the intermediate monocyte subset, derived from pseudobulking analysis.
6. (left) Heatmap showing the number of interactions from IgG4high-switched memory B cells (sender) to all myeloid cell subsets (receivers). (right) Heatmap showing the number of interactions from CD4^+^ Tfh cells (sender) to all myeloid cell subsets (receivers).
7. (left) Bar plot showing the information flow from IgG4high-switched memory B cells to intermediate monocytes. Right: Dot plot illustrating the specific ligand‒receptor communication probabilities. (right) Bar plot showing the information flow from CD4^+^ Tfh cells to intermediate monocytess. Right: Dot plot illustrating the specific ligand‒receptor communication probabilities.

**Fig S6. Multicolor Flow Cytometry Analysis of PBMCs from HC, CP, PDAC, and AIP Patient Cohorts.**

1. Flow cytometry gating strategy for IgG4high-switched memory B cells (CD45^+^CD19^+^CD27^+^CD38^+^IgD^-^CD23^+^), Tfh cells (CD45^+^CD3^+^CD4^+^CD45RA^-^PD1^+^CXCR5^+^) and intermediate monocytes (CD45^+^CD3^-^CD19^-^CD14^+^CD16^+^).
2. Box plots showing the proportional variations in CD19^+^ B cells, Plasmablasts， switched memory B cells, non-switched memory B cells, CD3^+^ T cells, CD4^+^ T helper cells and monocytes among HCs, patients with CP, patients with PDAC, and patients with AIP. Statistical differences in these proportions were determined using one-way ANOVA (**p<0.01, ***p<0.001, **** p< 0.0001).

**Fig S7. Diagnostic Performance of Tfh Cells, IgG4high-switched Memory B cells, Intermediate Monocytes, Plasmablasts and IgG4, and comparison of the Nomogram, IgG4, and Combined models.**

1. Comparison of Receiver operating characteristic (ROC) curves for proportions of Tfh cells, IgG4high-switched memory B cells and intermediate monocytes in differentiating AIP from HCs, CP and PDAC patients.
2. ROC curves of plasmablast proportions for differentiating AIP from HC, CP, and PDAC patients.
3. ROC curves of serum IgG4 levels for differentiating AIP from HC, CP, and PDAC patients.
4. Variance inflation factor (VIF) analysis of variables included in the logistic regression analyses.
5. ROC curves of the IgG4, 4-variable, and combined models.
6. Calibration plots of the IgG4, 4-variable, and combined models.
7. Decision curve analysis of the IgG4, 4-variable, and combined models.

**Fig S8. Prognosic value of IgG4high-Switched Memory B Cells, Tfh cells and Intermediate Monocytes in AIP patients.**

1. Bar plots showing the follow-up duration in the non-relapse and relapse groups of AIP patients. Statistical differences were determined by Mann-Whitney U-test.
2. Before-after plots illustrating the changes in the proportions of IgG4high-switched memory B cells and Tfh cells in each patient. Statistical differences were determined by paired t test.
3. Before-after plots illustrating the changes in IgG4 and in the proportions of IgG4high-switched memory B cells, Tfh cells and intermediate monocytes and in each patient. Statistical differences were determined by paired t test.
4. Violin plot showing the decrease rate of the proportions of IgG4high-switched memory B cells and intermediate monocytes in the non-relapse and relapse groups of AIP patients. Statistical differences were determined by unpaired t test

**SUPPLEMENTARY TABLES:**

| **Table S1. Clinical information of AIP and PDAC patients and** **healthy controls included for scRNA-seq.** | | | | | |
| --- | --- | --- | --- | --- | --- |
| **Group** | **AIP (n = 10)** | | **PDAC (n = 13)** | **HC (n=11)** | **P value** |
| **Age**  **(y/o, mean ± SD)** | 67.0 ± 8.7 | | 59.31 ± 9.3 | 43.09 ± 7.9 | <0.0001 |
| **Gender (male/female)** | 8/2 | | 7/6 | 5/6 | 0.3 |
| **Body mass index (kg/m^2^, mean ± SD)** | 21.0 ± 2.5 | | - | - | - |
| **IgG4**  **(g/L, mean** ± **SD)** | 7.9 ± 6.2 | | - | - | - |
| **Allergy history**  **(Yes/No)** | 0/10 | | - | - | - |
| **Other organ involvement** | **Bile duct and liver** | 7 | - | - |  |
|  | **Kidney** | 1 |  |  |  |
|  | **Salivary glands** | 1 |  |  |  |
|  | **Lymph nodes** | 8 |  |  |  |
|  | **Large blood vessels** | 1 |  |  |  |
|  | **Others** | 0 |  |  |  |
| **IgG**  **(g/, mean ± SD L)** | 16.2 ± 4.3 | | - | - | - |
| **IgE**  **(g/L, mean ± SD)** | 234.0 ± 327.0 | | - | - | - |
| **IgA**  **(g/L, mean ± SD)** | 1.8 ± 0.5 | | - | - | - |
| **IgM**  **(g/L, mean ± SD)** | 18.9 ± 37.0 | | - | - | - |
| **CRP**  **(mg/L, mean ± SD)** | 5.4 ± 5.8 | | - | - | - |
| **IgG4-RI**  **(mean ± SD** | 10.8 ± 6.9 | | - | - | - |

AIP, autoimmune pancreatitis; PDAC, pancreatic ductal adenocarcinoma; HC, healthy controls; SD, standard deviation; CRP, C-reactive protein; IgG4-RI, IgG4-related inflammation

| **Table S2. Baseline characteristics of AIP, CP, PDAC patients and healthy controls enrolled for multicolor flow cytometry analysis.** | | | | | |
| --- | --- | --- | --- | --- | --- |
| **Internal cohort** | | | | | |
| **Group** | **AIP (n = 71)** | **CP (n = 39)** | **PDAC (n = 45)** | **HC (n = 43)** | **P value** |
| **Age**  **(y/o, mean ± SD)** | 64.5 ± 12.1 | 61.3 ± 8.2 | 61.5 ± 8.4 | 46.2 ± 9.9 | <0.0001 |
| **Gender**  **(male/female)** | 58/13 | 31/7 | 32/13 | 29/14 | 0.2 |
| **Body mass index (kg/m^2^, mean±SD)** | 21.5 ± 1.8 | 21.9 ± 2.4 | 21.8 ± 1.9 | 21.9 ± 2.0 | 0.2 |
| **IgG4**  **(g/L, mean**±**SD)** | 9.5 ± 9.4 | 0.8 ± 0.7 | 0.7 ± 0.4 | 0.6 ± 0.4 | <0.0001 |
| **Allergy history**  **(Yes/No)** | 11/60 | 1/38 | 2/43 | 2/41 | 0.07 |
| **External cohort** | | | | | |
| **Group** | **AIP (n =25)** | **CP (n = 10)** | **PDAC (n = 10)** | **HC (n = 10)** | **P value** |
| **Age**  **(y/o, mean ± SD)** | 62.7 ± 12.6 | 54.8 ± 12.4 | 63.1 ± 7.534 | 57.4 ± 3.2 | 0.1 |
| **Gender (male/female)** | 21/4 | 5/5 | 8/2 | 8/2 | 0.3 |
| **Body mass index (kg/m^2^, mean±SD)** | 21.1 ± 1.0 | 21.5 ± 2.6 | 20.6 ± 3.4 | 21.9 ± 0.8 | 0.10 |
| **IgG4**  **(g/L, mean**±**SD)** | 7.3 ± 6.0 | 0.8 ± 0.6 | 0.7 ± 0.3 | 0.6 ± 0.3 | <0.0001 |
| **Allergy history**  **(Yes/No)** | 0/25 | 0/10 | 0/10 | 0/10 | - |

AIP, autoimmune pancreatitis; PDAC, pancreatic ductal adenocarcinoma; HC, healthy controls; **y/**o, years old**;** SD, standard deviation;

| \| **Table S3. Clinical characteristics of AIP, CP and PDAC patients enrolled for multicolor flow cytometry analysis.** \| \| \| \| \| \| --- \| --- \| --- \| --- \| --- \| \| **Internal cohort** \| \| \| \| \| \| \| **Group** \| **AIP (n = 71)** \| **CP (n = 39)** \| **PDAC (n = 45)** \| **P value** \| \| \| **IgE**  **(g/L, mean** ± **SD)** \| 293.8 ± 495.3 \| 57.0 ± 101.2 \| 62.5 ± 92.3 \| <0.0001 \| \| \| **Eosinophil Percentage**  **(%, mean ± SD)** \| 4.0 ± 3.1 \| 2.1 ± 1.5 \| 1.7 ± 1.3 \| <0.0001 \| \| \| **Absolute Eosinophil Count**  **(×10⁹/L, mean ± SD)** \| 0.2 ± 0.2 \| 0.1 ± 0.1 \| 0.1 ± 0.07 \| <0.0001 \| \| \| **CA19-9**  **(U/ml, mean ± SD)** \| 47.17 ± 112.5 \| 27.93 ± 57.7 \| 2969 ± 6375 \| 0.0004 \| \| \| **ALT**  **(IU/L, mean ± SD)** \| 90.2 ± 140.6 \| 21.2 ± 15.2 \| 86.0 ± 149.0 \| 0.009 \| \| \| **AST**  **(IU/L, mean ± SD)** \| 62.4 ± 86.3 \| 24.7 ± 10.5 \| 64.0 ± 100.2 \| 0.03 \| \| \| **ALP**  **(IU/L, mean ± SD)** \| 223.2 ± 278.1 \| 80.6 ± 28.6 \| 167.2 ± 196.2 \| 0.001 \| \| \| **γ-GT**  **(IU/L, mean ± SD)** \| 233.9 ± 338.7 \| 45.1 ± 70.1 \| 226.9 ± 359.3 \| 0.002 \| \| \| **TBIL**  **(μmol/L, mean ± SD)** \| 48.7 ± 69.8 \| 10.6 ± 4.8 \| 58.0 ± 86.2 \| 0.002 \| \| \| **DBIL**  **(μmol/L, mean ± SD)** \| 20.7 ± 38.5 \| 1.5 ± 0.9 \| 26.9 ± 47.1 \| 0.003 \| \| \| **ALB**  **(g/L, mean ± SD)** \| 36.6 ± 5.2 \| 40.2 ± 3.4 \| 39.1 ± 4.8 \| 0.0002 \| \| \| **AMY**  **(IU/L)** \| 123.4 ± 116.5 \| 103.5 ± 148.3 \| 105.9 ± 83.5 \| 0.6 \| \| \| **External cohort** \| \| \| \| \| \| \| **Group** \| **AIP (n =25)** \| **CP (n = 10)** \| **PDAC (n = 10)** \| **P value** \| \| \| **IgE**  **(g/L, mean** ± **SD)** \| 331.8 ± 382.5 \| 147.9 ± 262.1 \| 86.3 ± 48.7 \| 0.02 \| \| \| **Eosinophil Percentage (%, mean** ± **SD)** \| 3.4 ± 2.5 \| 2.9 ± 1.8 \| 3.7 ± 6.7 \| 0.9 \| \| \| **Absolute Eosinophil Count**  **(×10⁹/L, mean** ± **SD)** \| 0.2 ± 0.2 \| 0.2 ± 0.1 \| 0.3 ± 0.6 \| 0.8 \| \| \| **CA19-9**  **(U/ml, mean** ± **SD)** \| 38.2 ± 60.4 \| 140.7 ± 384.2 \| 417.4 ± 891.0 \| 0.3 \| \| \| **ALT**  **(IU/L, mean** ± **SD)** \| 46.0 ± 74.2 \| 25.6 ± 18.3 \| 106.2 ± 129.9 \| 0.1 \| \| \| **AST**  **(IU/L, mean** ± **SD)** \| 41.9 ± 40.5 \| 34.7 ± 28.7 \| 64.2 ± 70.6 \| 0.4 \| \| \| **ALP**  **(IU/L, mean** ± **SD)** \| 157.9 ± 133.5 \| 77.1 ± 23.4 \| 174.3 ± 153.5 \| 0.14 \| \| \| **γ-GT**  **(IU/L, mean** ± **SD)** \| 156.2 ± 297.0 \| 29.10 ± 22.92 \| 361.4 ± 567.2 \| 0.2 \| \| \| **TBIL**  **(μmol/L, mean** ± **SD)** \| 15.8 ± 12.2 \| 8.9 ± 2.0 \| 28.1 ± 21.7 \| 0.04 \| \| \| **DBIL**  **(μmol/L, mean** ± **SD)** \| 4.9 ± 7.2 \| 1.5 ± 0.5 \| 10.1 ± 11.8 \| 0.09 \| \| \| **ALB**  **(g/L, mean** ± **SD)** \| 39.0 ± 4.8 \| 37.2 ± 2.5 \| 38.5 ± 4.8 \| 0.5 \| \| \| **AMY**  **(IU/L, mean** ± **SD)** \| 99.4 ± 101.6 \| 213.8 ± 293.7 \| 93.2 ± 74.8 \| 0.3 \| \|   CA19-9, carbohydrate antigen 19-9; ALT, alanine aminotransferase; AST, aspartate aminotransferase; ALP, alkaline phosphatase; γ-GT, gamma-glutamyl transferase; TBIL, total bilirubin; DBIL, direct bilirubin; ALB, albumin; AMY, amylase |
| --- | --- | --- | --- | --- | --- | --- | --- | --- | --- | --- | --- | --- | --- | --- | --- | --- | --- | --- | --- | --- | --- | --- | --- | --- | --- | --- | --- | --- | --- | --- | --- | --- | --- | --- | --- | --- | --- | --- | --- | --- | --- | --- | --- | --- | --- | --- | --- | --- | --- | --- | --- | --- | --- | --- | --- | --- | --- | --- | --- | --- | --- | --- | --- | --- | --- | --- | --- | --- | --- | --- | --- | --- | --- | --- | --- | --- | --- | --- | --- | --- | --- | --- | --- | --- | --- | --- | --- | --- | --- | --- | --- | --- | --- | --- | --- | --- | --- | --- | --- | --- | --- | --- | --- | --- | --- | --- | --- | --- | --- | --- | --- | --- | --- | --- | --- | --- | --- | --- | --- | --- | --- | --- | --- | --- | --- | --- | --- | --- | --- | --- | --- | --- | --- | --- | --- | --- | --- | --- | --- | --- | --- | --- | --- | --- | --- | --- | --- | --- | --- | --- | --- | --- | --- | --- | --- | --- | --- | --- | --- | --- | --- | --- | --- | --- | --- | --- | --- | --- | --- | --- | --- | --- | --- |

| **Table S4. Top10 marker genes for main cell type.** | | | | | |
| --- | --- | --- | --- | --- | --- |
| **avg_log2FC** | **pct.1** | **pct.2** | **p_val_adj** | **cluster** | **gene** |
| 4.445988494 | 0.755 | 0.059 | 0 | T | *IL7R* |
| 3.847657637 | 0.618 | 0.065 | 0 | T | *TCF7* |
| 4.128279835 | 0.451 | 0.033 | 0 | T | *LEF1* |
| 4.337917078 | 0.413 | 0.025 | 0 | T | *MAL* |
| 4.257593052 | 0.411 | 0.027 | 0 | T | *CAMK4* |
| 4.077432284 | 0.391 | 0.03 | 0 | T | *AQP3* |
| 3.994350301 | 0.389 | 0.03 | 0 | T | *CD5* |
| 4.127793489 | 0.323 | 0.023 | 0 | T | *INPP4B* |
| 4.22616968 | 0.274 | 0.017 | 0 | T | *TRAT1* |
| 3.7478424 | 0.26 | 0.022 | 0 | T | *THEMIS* |
| 3.919117358 | 0.93 | 0.117 | 0 | NK | *GZMB* |
| 4.659744448 | 0.792 | 0.091 | 0 | NK | *SPON2* |
| 4.348598526 | 0.708 | 0.078 | 0 | NK | *CLIC3* |
| 5.059885924 | 0.644 | 0.03 | 0 | NK | *KLRF1* |
| 4.127380137 | 0.492 | 0.03 | 0 | NK | *TRDC* |
| 6.027265718 | 0.404 | 0.011 | 0 | NK | *SH2D1B* |
| 4.363742852 | 0.429 | 0.049 | 0 | NK | *IGFBP7* |
| 4.269352485 | 0.325 | 0.023 | 0 | NK | *KLRC2* |
| 4.116921138 | 0.291 | 0.02 | 0 | NK | *KLRC3* |
| 4.033507055 | 0.267 | 0.029 | 0 | NK | *MYOM2* |
| 9.243351448 | 0.73 | 0.002 | 0 | B | *LINC00926* |
| 8.909820974 | 0.535 | 0.002 | 0 | B | *FCRLA* |
| 9.004365013 | 0.532 | 0.002 | 0 | B | *CD19* |
| 9.03732532 | 0.526 | 0.003 | 0 | B | *FCER2* |
| 8.723467939 | 0.484 | 0.002 | 0 | B | *FCRL1* |
| 10.22028806 | 0.461 | 0 | 0 | B | *VPREB3* |
| 8.527379 | 0.428 | 0.002 | 0 | B | *CD22* |
| 9.483590311 | 0.387 | 0 | 0 | B | *CD24* |
| 8.504646899 | 0.351 | 0.001 | 0 | B | *IGHD* |
| 9.657727638 | 0.301 | 0 | 0 | B | *PAX5* |
| 7.881231106 | 0.804 | 0.023 | 0 | Plasma | *MZB1* |
| 8.166017488 | 0.774 | 0.033 | 0 | Plasma | *JCHAIN* |
| 8.902156592 | 0.473 | 0.016 | 0 | Plasma | *IGHG1* |
| 8.175075388 | 0.46 | 0.004 | 0 | Plasma | *TNFRSF17* |
| 9.477795396 | 0.344 | 0.018 | 0 | Plasma | *IGHA1* |
| 8.97854298 | 0.321 | 0.005 | 0 | Plasma | *IGHG4* |
| 7.90174173 | 0.298 | 0.017 | 0 | Plasma | *IGLC2* |
| 8.572230865 | 0.277 | 0.007 | 0 | Plasma | *IGLC1* |
| 7.97941546 | 0.277 | 0.011 | 0 | Plasma | *IGKV4-1* |
| 9.860186183 | 0.259 | 0.007 | 0 | Plasma | *IGHA2* |
| 6.977874322 | 0.959 | 0.068 | 0 | CD14^+^ Monocyte | *S100A8* |
| 6.710248212 | 0.962 | 0.098 | 0 | CD14^+^ Monocyte | *S100A9* |
| 7.313084502 | 0.842 | 0.018 | 0 | CD14^+^ Monocyte | *S100A12* |
| 6.1914254 | 0.85 | 0.03 | 0 | CD14^+^ Monocyte | *VCAN* |
| 6.149436771 | 0.734 | 0.021 | 0 | CD14^+^ Monocyte | *CD14* |
| 5.840587974 | 0.732 | 0.029 | 0 | CD14^+^ Monocyte | *CSF3R* |
| 5.681165374 | 0.455 | 0.016 | 0 | CD14^+^ Monocyte | *RBP7* |
| 5.758590742 | 0.317 | 0.01 | 0 | CD14^+^ Monocyte | *MGST1* |
| 5.759062014 | 0.29 | 0.009 | 0 | CD14^+^ Monocyte | *TREM1* |
| 6.96107967 | 0.252 | 0.003 | 0 | CD14^+^ Monocyte | *CLEC4E* |
| 7.983234292 | 0.706 | 0.011 | 0 | CD16^+^ Monocyte | *CDKN1C* |
| 4.732881356 | 0.635 | 0.029 | 0 | CD16^+^ Monocyte | *TCF7L2* |
| 6.770108659 | 0.471 | 0.005 | 0 | CD16^+^ Monocyte | *HES4* |
| 4.704601171 | 0.433 | 0.018 | 0 | CD16^+^ Monocyte | *CTSL* |
| 6.877975399 | 0.353 | 0.005 | 0 | CD16^+^ Monocyte | *CKB* |
| 4.928714824 | 0.351 | 0.012 | 0 | CD16^+^ Monocyte | *BATF3* |
| 5.292934219 | 0.337 | 0.009 | 0 | CD16^+^ Monocyte | *NEURL1* |
| 7.521651863 | 0.314 | 0.003 | 0 | CD16^+^ Monocyte | *C1QA* |
| 5.303676168 | 0.289 | 0.008 | 0 | CD16^+^ Monocyte | *MS4A4A* |
| 5.545203328 | 0.254 | 0.006 | 0 | CD16^+^ Monocyte | *ICAM4* |
| 6.481487468 | 0.739 | 0.017 | 0 | DC | *CLEC10A* |
| 8.753379649 | 0.646 | 0.003 | 0 | DC | *FCER1A* |
| 6.116340116 | 0.614 | 0.015 | 0 | DC | *CD1C* |
| 3.733242566 | 0.517 | 0.057 | 0 | DC | *HLA-DQA2* |
| 7.430085356 | 0.452 | 0.004 | 0 | DC | *ENHO* |
| 3.617755477 | 0.386 | 0.02 | 0 | DC | *NDRG2* |
| 5.07800903 | 0.37 | 0.008 | 0 | DC | *FLT3* |
| 3.70814448 | 0.283 | 0.014 | 0 | DC | *UPK3A* |
| 5.637421723 | 0.264 | 0.003 | 0 | DC | *CLIC2* |
| 4.366309792 | 0.251 | 0.007 | 0 | DC | *CYP2S1* |
| 11.31017457 | 0.76 | 0.001 | 0 | pDC | *CLEC4C* |
| 15.09167397 | 0.69 | 0 | 0 | pDC | *LRRC26* |
| 14.12612704 | 0.652 | 0 | 0 | pDC | *SCT* |
| 10.51082631 | 0.649 | 0.001 | 0 | pDC | *PTCRA* |
| 13.37345773 | 0.409 | 0 | 0 | pDC | *SHD* |
| 14.76944669 | 0.37 | 0 | 0 | pDC | *AC097375.1* |
| 10.73350175 | 0.302 | 0 | 0 | pDC | *KCNK17* |
| 10.38719228 | 0.292 | 0 | 0 | pDC | *ASIP* |
| 10.57005279 | 0.277 | 0 | 0 | pDC | *PROC* |
| 11.17996111 | 0.256 | 0 | 0 | pDC | *CUX2* |
| 9.41663495 | 0.498 | 0.002 | 0 | Prolifer | *TYMS* |
| 7.647159515 | 0.482 | 0.005 | 0 | Prolifer | *MKI67* |
| 7.395025546 | 0.461 | 0.006 | 0 | Prolifer | *RRM2* |
| 7.511867298 | 0.444 | 0.005 | 0 | Prolifer | *PCLAF* |
| 6.911172182 | 0.351 | 0.005 | 0 | Prolifer | *TK1* |
| 6.686322217 | 0.314 | 0.004 | 0 | Prolifer | *GINS2* |
| 7.1215458 | 0.311 | 0.003 | 0 | Prolifer | *CDT1* |
| 8.348357486 | 0.278 | 0.001 | 0 | Prolifer | *UHRF1* |
| 8.395603136 | 0.271 | 0.001 | 0 | Prolifer | *BIRC5* |
| 9.318439149 | 0.251 | 0 | 0 | Prolifer | *PKMYT1* |

| **Table S5. Top 10 marker genes for B cell subcluster.** | | | | | |
| --- | --- | --- | --- | --- | --- |
| **avg_log2FC** | **pct.1** | **pct.2** | **p_val_adj** | **cluster** | **gene** |
| 4.845084625 | 0.687 | 0.044 | 0 | Naive B | *TCL1A* |
| 2.455915695 | 0.779 | 0.264 | 0 | Naive B | *FCER2* |
| 3.221038285 | 0.578 | 0.096 | 0 | Naive B | *IL4R* |
| 2.183540313 | 0.66 | 0.255 | 0 | Naive B | *PLPP5* |
| 1.935850233 | 0.538 | 0.15 | 0 | Naive B | *IGHD* |
| 2.716353868 | 0.457 | 0.119 | 0 | Naive B | *YBX3* |
| 2.066339104 | 0.514 | 0.188 | 0 | Naive B | *BACH2* |
| 1.905331035 | 0.371 | 0.147 | 1.33E-258 | Naive B | *TSPAN13* |
| 2.283140302 | 0.283 | 0.077 | 2.10E-255 | Naive B | *APLP2* |
| 2.217952963 | 0.278 | 0.088 | 8.65E-218 | Naive B | *COL19A1* |
| 2.458799141 | 0.753 | 0.283 | 0 | Memory B | *CRIP1* |
| 2.331651863 | 0.513 | 0.107 | 0 | Memory B | *ANXA2* |
| 3.434549973 | 0.448 | 0.042 | 0 | Memory B | *TNFRSF13B* |
| 2.967230992 | 0.399 | 0.036 | 0 | Memory B | *CD27* |
| 4.090959271 | 0.379 | 0.023 | 0 | Memory B | *AIM2* |
| 2.992230047 | 0.415 | 0.061 | 0 | Memory B | *ITGB1* |
| 2.782728485 | 0.394 | 0.051 | 0 | Memory B | *LGALS1* |
| 2.129348185 | 0.426 | 0.097 | 0 | Memory B | *ACP5* |
| 3.175005644 | 0.351 | 0.049 | 0 | Memory B | *LINC01781* |
| 3.902459489 | 0.304 | 0.021 | 0 | Memory B | *CLECL1P* |
| 6.742596213 | 0.359 | 0.008 | 0 | Plasmablast | *AQP3* |
| 5.394581169 | 0.313 | 0.01 | 0 | Plasmablast | *SLAMF7* |
| 5.636864679 | 0.255 | 0.007 | 0 | Plasmablast | *SPN* |
| 4.631629225 | 0.389 | 0.034 | 2.15E-278 | Plasmablast | *TNFRSF17* |
| 4.835042495 | 0.51 | 0.067 | 1.05E-271 | Plasmablast | *FKBP11* |
| 4.674864417 | 0.632 | 0.185 | 3.52E-166 | Plasmablast | *MZB1* |
| 4.639410379 | 0.639 | 0.315 | 1.21E-85 | Plasmablast | *JCHAIN* |
| 5.11866816 | 0.287 | 0.078 | 2.24E-50 | Plasmablast | *IGLC1* |
| 5.394419596 | 0.384 | 0.167 | 1.27E-38 | Plasmablast | *IGHG1* |
| 6.035177167 | 0.31 | 0.206 | 3.71E-07 | Plasmablast | *IGHA1* |

| **Table S6. Top 10 marker genes for Memory B cell subcluster.** | | | | | | | | | |
| --- | --- | --- | --- | --- | --- | --- | --- | --- | --- |
| **avg_log2FC** | **pct.1** | **pct.2** | | **p_val_adj** | | **cluster** | | | **gene** |
| 1.461420159 | 0.573 | 0.308 | | 5.22E-130 | | Non-switched memory B | | | *CD69* |
| 1.138219571 | 0.552 | 0.277 | | 1.27E-113 | | Non-switched memory B | | | *VPREB3* |
| 1.367292873 | 0.381 | 0.192 | | 2.21E-70 | | Non-switched memory B | | | *LINC01857* |
| 0.912879045 | 0.516 | 0.313 | | 8.63E-65 | | Non-switched memory B | | | *ALOX5* |
| 1.195960413 | 0.335 | 0.169 | | 2.95E-53 | | Non-switched memory B | | | *PMAIP1* |
| 1.387121939 | 0.277 | 0.133 | | 1.89E-48 | | Non-switched memory B | | | *CCR7* |
| 0.905535837 | 0.383 | 0.208 | | 1.65E-47 | | Non-switched memory B | | | *ITM2C* |
| 0.898958545 | 0.412 | 0.257 | | 1.36E-41 | | Non-switched memory B | | | *PDE4B* |
| 1.062487656 | 0.305 | 0.175 | | 1.93E-34 | | Non-switched memory B | | | *MYC* |
| 1.035153927 | 0.257 | 0.132 | | 2.92E-34 | | Non-switched memory B | | | *TENT5C* |
| 1.618250984 | 0.729 | 0.437 | | 4.83E-177 | | Switched memory B | | | *S100A10* |
| 2.564519479 | 0.374 | 0.097 | | 3.90E-167 | | Switched memory B | | | *CRIP2* |
| 1.631647033 | 0.597 | 0.342 | | 1.20E-134 | | Switched memory B | | | *ITGB1* |
| 2.122777334 | 0.295 | 0.078 | | 8.99E-118 | | Switched memory B | | | *IGHA2* |
| 2.140392659 | 0.325 | 0.101 | | 1.99E-114 | | Switched memory B | | | *HIPK2* |
| 1.466545519 | 0.373 | 0.13 | | 1.16E-106 | | Switched memory B | | | *PDE4D* |
| 1.53913484 | 0.338 | 0.134 | | 4.33E-82 | | Switched memory B | | | *SSPN* |
| 1.52140909 | 0.324 | 0.126 | | 3.23E-79 | | Switched memory B | | | *COCH* |
| 1.567912277 | 0.253 | 0.091 | | 3.32E-65 | | Switched memory B | | | *GSN* |
| 1.571268645 | 0.266 | 0.103 | | 7.07E-63 | | Switched memory B | | | *GRAMD1C* |
| 3.784709484 | 0.441 | 0.035 | | 4.99E-278 | | IgG4high-switched memory B | | | *IGHE* |
| 2.444575218 | 0.507 | 0.102 | | 5.61E-152 | | IgG4high-switched memory B | | | *HOPX* |
| 3.272352018 | 0.417 | 0.072 | | 3.16E-149 | | IgG4high-switched memory B | | | *IL4R* |
| 2.140097143 | 0.572 | 0.167 | | 1.43E-117 | | IgG4high-switched memory B | | | *PDE4D* |
| 2.013195025 | 0.645 | 0.244 | | 3.77E-103 | | IgG4high-switched memory B | | | *FCER2* |
| 1.863465945 | 0.563 | 0.218 | | 4.01E-80 | | IgG4high-switched memory B | | | *PLPP5* |
| 1.877226437 | 0.473 | 0.158 | | 9.46E-73 | | IgG4high-switched memory B | | | *COCH* |
| 2.421456509 | 0.265 | 0.068 | | 4.57E-55 | | IgG4high-switched memory B | | | *IGHG4* |
| 2.037348561 | 0.341 | 0.108 | | 2.17E-53 | | IgG4high-switched memory B | | | *RHOC* |
| 1.894044098 | 0.326 | 0.123 | | 4.97E-38 | | IgG4high-switched memory B | | | *RNGTT* |
| 1.965889231 | 0.5 | 0.157 | | 5.76E-83 | | Atypical memory B | | | *CD1C* |
| 1.839399657 | 0.518 | 0.179 | | 1.44E-75 | | Atypical memory B | | | *HCK* |
| 1.562593822 | 0.535 | 0.193 | | 2.58E-68 | | Atypical memory B | | | *FGR* |
| 1.644647017 | 0.518 | 0.207 | | 2.95E-58 | | Atypical memory B | | | *PPP1R14A* |
| 1.709132451 | 0.307 | 0.088 | | 9.68E-49 | | Atypical memory B | | | *TUBB6* |
| 2.051163178 | 0.285 | 0.081 | | 2.98E-48 | | Atypical memory B | | | *CXCR3* |
| 1.718615569 | 0.268 | 0.075 | | 8.74E-43 | | Atypical memory B | | | *PTPRJ* |
| 1.680323641 | 0.369 | 0.148 | | 4.70E-37 | | Atypical memory B | | | *KLK1* |
| 1.597310294 | 0.283 | 0.102 | | 2.02E-30 | | Atypical memory B | | | *SIGLEC6* |
| 1.460916033 | 0.254 | 0.097 | | 8.47E-23 | | Atypical memory B | | | *CNFN* |
| 2.868185522 | 0.755 | 0.169 | | 1.14E-268 | | ABC | | | *FGR* |
| 4.914261278 | 0.287 | 0.014 | | 7.41E-241 | | ABC | | | *LILRB2* |
| 3.310891643 | 0.624 | 0.123 | | 5.18E-236 | | ABC | | | *FCRL5* |
| 3.45515776 | 0.45 | 0.069 | | 2.57E-193 | | ABC | | | *MPHOSPH6* |
| 4.011196451 | 0.283 | 0.024 | | 4.25E-174 | | ABC | | | *DTX1* |
| 4.027832817 | 0.268 | 0.021 | | 7.20E-170 | | ABC | | | *MACROD2* |
| 3.303746404 | 0.369 | 0.051 | | 1.43E-164 | | ABC | | | *PCDH9* |
| 3.183971525 | 0.329 | 0.05 | | 9.47E-133 | | ABC | | | *ITGAX* |
| 3.105979481 | 0.252 | 0.04 | | 3.40E-94 | | ABC | | | *ARL4D* |
| 2.912465309 | 0.264 | 0.046 | | 6.87E-92 | | ABC | | | *TENT5A* |
| **Table S7. Top 10 marker genes for CD4^+^ T cell subcluster.** | | | | | | | | | |
| **avg_log2FC** | **pct.1** | | **pct.2** | | **p_val_adj** | | **cluster** | **gene** | |
| 1.648850251 | 0.703 | | 0.324 | | 0 | | CD4 T Naive | *CCR7* | |
| 1.098969509 | 0.755 | | 0.466 | | 0 | | CD4 T Naive | *LEF1* | |
| 1.948885261 | 0.348 | | 0.109 | | 0 | | CD4 T Naive | *ACTN1* | |
| 2.929294444 | 0.266 | | 0.042 | | 0 | | CD4 T Naive | *AIF1* | |
| 1.336024422 | 0.395 | | 0.192 | | 0 | | CD4 T Naive | *NUCB2* | |
| 1.342527735 | 0.386 | | 0.184 | | 0 | | CD4 T Naive | *SATB1* | |
| 1.174079419 | 0.388 | | 0.203 | | 0 | | CD4 T Naive | *TRABD2A* | |
| 1.70448686 | 0.262 | | 0.094 | | 0 | | CD4 T Naive | *CHRM3-AS2* | |
| 1.067105825 | 0.399 | | 0.233 | | 0 | | CD4 T Naive | *TGFBR2* | |
| 1.263333519 | 0.267 | | 0.122 | | 0 | | CD4 T Naive | *TSHZ2* | |
| 0.783457977 | 0.901 | | 0.818 | | 0 | | CD4 T memory | *JUNB* | |
| 0.913840535 | 0.428 | | 0.25 | | 1.42E-274 | | CD4 T memory | *LIMS1* | |
| 1.207235187 | 0.258 | | 0.122 | | 1.98E-250 | | CD4 T memory | *PASK* | |
| 0.691699882 | 0.525 | | 0.365 | | 6.42E-204 | | CD4 T memory | *TRADD* | |
| 0.631845746 | 0.548 | | 0.412 | | 9.28E-144 | | CD4 T memory | *FOS* | |
| 0.658265659 | 0.414 | | 0.291 | | 3.21E-124 | | CD4 T memory | *GPR183* | |
| 0.586183903 | 0.394 | | 0.273 | | 1.37E-115 | | CD4 T memory | *ARID5B* | |
| 0.60047779 | 0.428 | | 0.31 | | 3.10E-113 | | CD4 T memory | *SLC2A3* | |
| 0.776904839 | 0.277 | | 0.191 | | 2.00E-79 | | CD4 T memory | *SOCS3* | |
| 0.592924735 | 0.264 | | 0.186 | | 1.79E-63 | | CD4 T memory | *CDC14A* | |
| 3.127448226 | 0.583 | | 0.124 | | 0 | | CD4 *ISG*^+^ T | *MX1* | |
| 4.159103026 | 0.41 | | 0.035 | | 0 | | CD4 *ISG*^+^ T | *IFI44L* | |
| 3.386993174 | 0.413 | | 0.057 | | 0 | | CD4 *ISG*^+^ T | *OAS1* | |
| 3.208544968 | 0.281 | | 0.039 | | 0 | | CD4 *ISG*^+^ T | *OAS3* | |
| 2.380172674 | 0.507 | | 0.138 | | 6.32E-273 | | CD4 *ISG*^+^ T | *IRF7* | |
| 2.826751636 | 0.56 | | 0.177 | | 1.36E-272 | | CD4 *ISG*^+^ T | *IFI6* | |
| 2.586909669 | 0.609 | | 0.222 | | 4.13E-249 | | CD4 *ISG*^+^ T | *ISG15* | |
| 2.335978487 | 0.446 | | 0.116 | | 8.84E-248 | | CD4 *ISG*^+^ T | *EPSTI1* | |
| 2.327155485 | 0.301 | | 0.074 | | 8.43E-166 | | CD4 *ISG*^+^ T | *SAMD9L* | |
| 2.307898431 | 0.276 | | 0.069 | | 2.48E-149 | | CD4 *ISG*^+^ T | *HERC5* | |
| 1.642404277 | 0.603 | | 0.253 | | 0 | | CD4 Tfh | *LIMS1* | |
| 1.064843906 | 0.72 | | 0.463 | | 0 | | CD4 Tfh | *ITM2A* | |
| 1.797885437 | 0.337 | | 0.128 | | 0 | | CD4 Tfh | *PASK* | |
| 1.534563544 | 0.287 | | 0.097 | | 1.18E-286 | | CD4 Tfh | *LGALS9* | |
| 1.455503085 | 0.285 | | 0.097 | | 2.62E-278 | | CD4 Tfh | *TIGIT* | |
| 1.078089942 | 0.649 | | 0.398 | | 2.67E-278 | | CD4 Tfh | *CORO1B* | |
| 1.062670738 | 0.438 | | 0.227 | | 3.58E-195 | | CD4 Tfh | *KLRB1* | |
| 1.094763802 | 0.48 | | 0.276 | | 3.48E-179 | | CD4 Tfh | *MT2A* | |
| 1.202616738 | 0.267 | | 0.111 | | 1.17E-172 | | CD4 Tfh | *TRIB2* | |
| 1.101251324 | 0.264 | | 0.123 | | 1.34E-129 | | CD4 Tfh | *ICOS* | |
| 1.577038078 | 0.61 | | 0.242 | | 0 | | CD4 *TIMP1*^+^ T | *LGALS1* | |
| 2.283117841 | 0.317 | | 0.068 | | 0 | | CD4 *TIMP1*^+^ T | *CCR6* | |
| 2.178308678 | 0.322 | | 0.08 | | 0 | | CD4 *TIMP1*^+^ T | *CAPG* | |
| 2.135838164 | 0.307 | | 0.073 | | 0 | | CD4 *TIMP1*^+^ T | *LGALS3* | |
| 2.704423617 | 0.279 | | 0.046 | | 0 | | CD4 *TIMP1*^+^ T | *CTSH* | |
| 1.543781988 | 0.313 | | 0.105 | | 0 | | CD4 *TIMP1*^+^ T | *TNFRSF4* | |
| 2.336962547 | 0.255 | | 0.052 | | 0 | | CD4 *TIMP1*^+^ T | *LMNA* | |
| 2.373032191 | 0.254 | | 0.053 | | 0 | | CD4 *TIMP1*^+^ T | *WDR86-AS1* | |
| 1.492197882 | 0.262 | | 0.095 | | 0 | | CD4 *TIMP1*^+^ T | *PDE4D* | |
| 1.479873018 | 0.251 | | 0.088 | | 0 | | CD4 *TIMP1*^+^ T | *NSG1* | |
| 1.872231735 | 0.718 | | 0.125 | | 0 | | CD4 T effector memory | *CCL5* | |
| 4.537971447 | 0.595 | | 0.036 | | 0 | | CD4 T effector memory | *GZMK* | |
| 1.915242128 | 0.549 | | 0.107 | | 0 | | CD4 T effector memory | *GZMA* | |
| 2.364967976 | 0.507 | | 0.129 | | 0 | | CD4 T effector memory | *LYAR* | |
| 1.543470802 | 0.339 | | 0.1 | | 0 | | CD4 T effector memory | *HOPX* | |
| 1.798249936 | 0.422 | | 0.188 | | 0 | | CD4 T effector memory | *DUSP2* | |
| 1.849537638 | 0.322 | | 0.092 | | 0 | | CD4 T effector memory | *CXCR3* | |
| 1.577242791 | 0.334 | | 0.119 | | 0 | | CD4 T effector memory | *KLRG1* | |
| 1.580074088 | 0.329 | | 0.12 | | 0 | | CD4 T effector memory | *STOM* | |
| 1.594007936 | 0.253 | | 0.098 | | 5.22E-280 | | CD4 T effector memory | *NSG1* | |
| 6.234149061 | 0.539 | | 0.013 | | 0 | | CD4 Treg | *FOXP3* | |
| 2.892883371 | 0.454 | | 0.081 | | 0 | | CD4 Treg | *TIGIT* | |
| 2.543167588 | 0.51 | | 0.142 | | 0 | | CD4 Treg | *HLA-DRB1* | |
| 5.139056533 | 0.375 | | 0.013 | | 0 | | CD4 Treg | *IKZF2* | |
| 4.886450621 | 0.342 | | 0.018 | | 0 | | CD4 Treg | *RTKN2* | |
| 2.731140914 | 0.395 | | 0.085 | | 0 | | CD4 Treg | *STAM* | |
| 2.858868926 | 0.385 | | 0.077 | | 0 | | CD4 Treg | *CTLA4* | |
| 2.721144064 | 0.322 | | 0.067 | | 0 | | CD4 Treg | *HLA-DRA* | |
| 3.417048658 | 0.285 | | 0.041 | | 0 | | CD4 Treg | *IL2RA* | |
| 2.547826313 | 0.266 | | 0.06 | | 0 | | CD4 Treg | *RGS1* | |
| 7.644508884 | 0.951 | | 0.014 | | 0 | | CD4 TEMRA | *GZMH* | |
| 6.567027416 | 0.972 | | 0.064 | | 0 | | CD4 TEMRA | *NKG7* | |
| 8.114668842 | 0.812 | | 0.005 | | 0 | | CD4 TEMRA | *FGFBP2* | |
| 6.282561123 | 0.797 | | 0.03 | | 0 | | CD4 TEMRA | *GNLY* | |
| 6.907643526 | 0.579 | | 0.007 | | 0 | | CD4 TEMRA | *GZMB* | |
| 6.993472101 | 0.47 | | 0.005 | | 0 | | CD4 TEMRA | *CX3CR1* | |
| 6.410787802 | 0.437 | | 0.006 | | 0 | | CD4 TEMRA | *CCL4* | |
| 7.109668993 | 0.429 | | 0.003 | | 0 | | CD4 TEMRA | *ADGRG1* | |
| 6.38752343 | 0.36 | | 0.004 | | 0 | | CD4 TEMRA | *FCRL6* | |
| 6.974609407 | 0.294 | | 0.002 | | 0 | | CD4 TEMRA | *PRSS23* | |

| **Table S8. Top 10 marker genes for CD8^+^ T cell subcluster.** | | | | | |
| --- | --- | --- | --- | --- | --- |
| **avg_log2FC** | **pct.1** | **pct.2** | **p_val_adj** | **cluster** | **gene** |
| 3.051630291 | 0.598 | 0.103 | 0 | CD8 T Naive | *CCR7* |
| 2.608344535 | 0.63 | 0.15 | 0 | CD8 T Naive | *LEF1* |
| 3.629894532 | 0.416 | 0.042 | 0 | CD8 T Naive | *ACTN1* |
| 2.589944996 | 0.448 | 0.078 | 0 | CD8 T Naive | *MAL* |
| 3.027808039 | 0.423 | 0.06 | 0 | CD8 T Naive | *AIF1* |
| 2.393482063 | 0.367 | 0.076 | 0 | CD8 T Naive | *TRABD2A* |
| 2.663300937 | 0.299 | 0.054 | 0 | CD8 T Naive | *ARMH1* |
| 2.549466556 | 0.286 | 0.05 | 0 | CD8 T Naive | *SPINT2* |
| 2.003622113 | 0.306 | 0.082 | 0 | CD8 T Naive | *MYC* |
| 1.998371511 | 0.302 | 0.084 | 0 | CD8 T Naive | *SH3YL1* |
| 1.746103164 | 0.729 | 0.273 | 0 | CD8 T memory | *GZMK* |
| 1.428395705 | 0.85 | 0.462 | 0 | CD8 T memory | *IL7R* |
| 1.190687465 | 0.659 | 0.408 | 0 | CD8 T memory | *FOS* |
| 1.893406168 | 0.275 | 0.08 | 0 | CD8 T memory | *GPR183* |
| 1.158176444 | 0.919 | 0.816 | 0 | CD8 T memory | *JUNB* |
| 1.390088774 | 0.315 | 0.131 | 2.18E-290 | CD8 T memory | *CXCR3* |
| 1.158496582 | 0.437 | 0.232 | 6.67E-270 | CD8 T memory | *CLDND1* |
| 1.042000583 | 0.378 | 0.19 | 1.43E-234 | CD8 T memory | *NELL2* |
| 1.120138531 | 0.264 | 0.129 | 1.28E-162 | CD8 T memory | *NSG1* |
| 0.987408801 | 0.364 | 0.212 | 5.87E-158 | CD8 T memory | *TRADD* |
| 1.240445804 | 0.589 | 0.276 | 0 | CD8 HLA-DRhigh T memory | *GZMK* |
| 1.303134364 | 0.648 | 0.343 | 0 | CD8 HLA-DRhigh T memory | *CMC1* |
| 1.109951396 | 0.724 | 0.45 | 0 | CD8 HLA-DRhigh T memory | *HLA-DRB1* |
| 1.694992529 | 0.463 | 0.197 | 0 | CD8 HLA-DRhigh T memory | *HLA-DRA* |
| 1.297163176 | 0.419 | 0.207 | 0 | CD8 HLA-DRhigh T memory | *HLA-DQA1* |
| 1.013972939 | 0.474 | 0.268 | 0 | CD8 HLA-DRhigh T memory | *HLA-DRB5* |
| 1.481960149 | 0.323 | 0.133 | 0 | CD8 HLA-DRhigh T memory | *HLA-DQB1* |
| 1.201368098 | 0.941 | 0.86 | 0 | CD8 HLA-DRhigh T memory | *CD74* |
| 1.312224157 | 0.294 | 0.132 | 6.05E-281 | CD8 HLA-DRhigh T memory | *HLA-DMA* |
| 1.050129229 | 0.553 | 0.391 | 2.74E-265 | CD8 HLA-DRhigh T memory | *COTL1* |
| 3.745682537 | 0.646 | 0.075 | 0 | CD8 KIR^+^ TEMRA | *TYROBP* |
| 2.134107051 | 0.733 | 0.22 | 0 | CD8 KIR^+^ TEMRA | *FCGR3A* |
| 2.70558919 | 0.512 | 0.096 | 0 | CD8 KIR^+^ TEMRA | *KLRF1* |
| 3.206780064 | 0.486 | 0.071 | 0 | CD8 KIR^+^ TEMRA | *KLRC2* |
| 2.803357138 | 0.394 | 0.064 | 0 | CD8 KIR^+^ TEMRA | *KLRC3* |
| 4.48910468 | 0.341 | 0.018 | 0 | CD8 KIR^+^ TEMRA | *TRDC* |
| 3.739336972 | 0.312 | 0.034 | 0 | CD8 KIR^+^ TEMRA | *TRDV1* |
| 3.391611945 | 0.303 | 0.032 | 0 | CD8 KIR^+^ TEMRA | *KIR3DL2* |
| 2.619992781 | 0.311 | 0.054 | 0 | CD8 KIR^+^ TEMRA | *IKZF2* |
| 3.45440047 | 0.276 | 0.028 | 0 | CD8 KIR^+^ TEMRA | *KIR2DL3* |
| 2.185813841 | 0.916 | 0.289 | 0 | CD8 TEMRA | *FGFBP2* |
| 1.555541068 | 0.878 | 0.315 | 0 | CD8 TEMRA | *GNLY* |
| 1.689908286 | 0.858 | 0.306 | 0 | CD8 TEMRA | *GZMB* |
| 1.562447071 | 0.822 | 0.387 | 0 | CD8 TEMRA | *LGALS1* |
| 1.812893627 | 0.705 | 0.292 | 0 | CD8 TEMRA | *ITGB1* |
| 2.215666436 | 0.506 | 0.124 | 0 | CD8 TEMRA | *PRSS23* |
| 1.503685126 | 0.62 | 0.238 | 0 | CD8 TEMRA | *ADGRG1* |
| 2.953821999 | 0.413 | 0.066 | 0 | CD8 TEMRA | *ZNF683* |
| 1.722035518 | 0.551 | 0.214 | 0 | CD8 TEMRA | *SPON2* |
| 2.389676322 | 0.256 | 0.053 | 0 | CD8 TEMRA | *ASCL2* |
| 3.114274854 | 0.939 | 0.245 | 0 | MAIT | *KLRB1* |
| 3.876954486 | 0.641 | 0.052 | 0 | MAIT | *TRAV1-2* |
| 5.061624438 | 0.574 | 0.032 | 0 | MAIT | *CEBPD* |
| 9.73782277 | 0.507 | 0.001 | 0 | MAIT | *SLC4A10* |
| 7.859294364 | 0.313 | 0.002 | 0 | MAIT | *LTK* |
| 5.427081897 | 0.319 | 0.01 | 0 | MAIT | *ZBTB16* |
| 7.965270529 | 0.265 | 0.002 | 0 | MAIT | *RORC* |
| 3.038122589 | 0.307 | 0.046 | 0 | MAIT | *DPP4* |
| 3.284096666 | 0.293 | 0.042 | 0 | MAIT | *LST1* |
| 5.954874876 | 0.257 | 0.006 | 0 | MAIT | *CCR6* |

| **Table S9. Top 10 marker genes for NK cell subcluster.** | | | | | |
| --- | --- | --- | --- | --- | --- |
| **avg_log2FC** | **pct.1** | **pct.2** | **p_val_adj** | **cluster** | **gene** |
| 2.1091888 | 0.858 | 0.304 | 0 | CD56^dim^*FCER1G*^+^ NK | *FCER1G* |
| 2.731380771 | 0.346 | 0.067 | 0 | CD56^dim^*FCER1G*^+^ NK | *TMIGD2* |
| 1.397019945 | 0.494 | 0.221 | 0 | CD56^dim^*FCER1G*^+^ NK | *C1orf162* |
| 1.508681205 | 0.455 | 0.199 | 0 | CD56^dim^*FCER1G*^+^ NK | *CEBPD* |
| 1.315169467 | 0.455 | 0.246 | 0 | CD56^dim^*FCER1G*^+^ NK | *CD160* |
| 1.860891458 | 0.309 | 0.1 | 0 | CD56^dim^*FCER1G*^+^ NK | *GSN* |
| 1.609147406 | 0.321 | 0.132 | 0 | CD56^dim^*FCER1G*^+^ NK | *CHST2* |
| 1.367220146 | 0.256 | 0.097 | 0 | CD56^dim^*FCER1G*^+^ NK | *AREG* |
| 1.883371422 | 0.287 | 0.13 | 0 | CD56^dim^*FCER1G*^+^ NK | *PTGDS* |
| 1.345327276 | 0.31 | 0.155 | 0 | CD56^dim^*FCER1G*^+^ NK | *CD38* |
| 1.73986592 | 0.689 | 0.312 | 0 | CD56^dim^*FCER1G*^-^ NK | *HLA-DRB1* |
| 2.176035059 | 0.55 | 0.186 | 0 | CD56^dim^*FCER1G*^-^ NK | *KLRC2* |
| 1.885403776 | 0.609 | 0.269 | 0 | CD56^dim^*FCER1G*^-^ NK | *CD3E* |
| 4.515228023 | 0.335 | 0.019 | 0 | CD56^dim^*FCER1G*^-^ NK | *LAG3* |
| 3.67752474 | 0.326 | 0.032 | 0 | CD56^dim^*FCER1G*^-^ NK | *PTMS* |
| 2.637041867 | 0.365 | 0.075 | 0 | CD56^dim^*FCER1G*^-^ NK | *TRG-AS1* |
| 1.816583273 | 0.312 | 0.102 | 0 | CD56^dim^*FCER1G*^-^ NK | *KLRC4* |
| 2.214226889 | 0.256 | 0.063 | 0 | CD56^dim^*FCER1G*^-^ NK | *HLA-DQA1* |
| 1.969455612 | 0.272 | 0.081 | 0 | CD56^dim^*FCER1G*^-^ NK | *LGALS3* |
| 1.735079519 | 0.29 | 0.102 | 0 | CD56^dim^*FCER1G*^-^ NK | *HLA-DRA* |
| 5.097013805 | 0.691 | 0.031 | 0 | CD56^bright^ NK | *GZMK* |
| 2.993275456 | 0.884 | 0.25 | 0 | CD56^bright^ NK | *SELL* |
| 3.679642373 | 0.695 | 0.112 | 0 | CD56^bright^ NK | *XCL1* |
| 4.242940895 | 0.609 | 0.063 | 0 | CD56^bright^ NK | *COTL1* |
| 2.939619857 | 0.558 | 0.079 | 0 | CD56^bright^ NK | *TCF7* |
| 5.913852965 | 0.457 | 0.012 | 0 | CD56^bright^ NK | *CAPG* |
| 7.302896909 | 0.393 | 0.003 | 0 | CD56^bright^ NK | *SPTSSB* |
| 2.954196469 | 0.422 | 0.055 | 0 | CD56^bright^ NK | *IL7R* |
| 7.716244216 | 0.297 | 0.002 | 0 | CD56^bright^ NK | *IGFBP4* |
| 4.189839406 | 0.266 | 0.017 | 0 | CD56^bright^ NK | *GPR183* |

| **Table S10. Top 10 marker genes for myeloid cell subcluster.** | | | | | |
| --- | --- | --- | --- | --- | --- |
| **avg_log2FC** | **pct.1** | **pct.2** | **p_val_adj** | **cluster** | **gene** |
| 1.82293533 | 0.904 | 0.395 | 0 | Classical Monocyte | *S100A12* |
| 1.246298026 | 0.903 | 0.507 | 0 | Classical Monocyte | *VCAN* |
| 1.659931858 | 0.997 | 0.735 | 0 | Classical Monocyte | *S100A8* |
| 1.260469712 | 0.505 | 0.281 | 0 | Classical Monocyte | *RBP7* |
| 1.231060501 | 0.424 | 0.216 | 0 | Classical Monocyte | *ITGAM* |
| 1.402012949 | 0.369 | 0.184 | 0 | Classical Monocyte | *SLC2A3* |
| 1.336259081 | 0.366 | 0.181 | 0 | Classical Monocyte | *MGST1* |
| 1.232208087 | 0.377 | 0.194 | 0 | Classical Monocyte | *IER3* |
| 1.219453592 | 0.268 | 0.113 | 0 | Classical Monocyte | *CLEC4E* |
| 1.576017299 | 0.999 | 0.886 | 0 | Classical Monocyte | *S100A9* |
| 1.142556952 | 0.974 | 0.751 | 0 | Intermediate Monocyte | *HLA-DPB1* |
| 1.151349419 | 0.666 | 0.386 | 3.63E-220 | Intermediate Monocyte | *PLAC8* |
| 1.538852533 | 0.553 | 0.287 | 7.75E-216 | Intermediate Monocyte | *APOBEC3A* |
| 1.154722489 | 0.54 | 0.27 | 1.81E-195 | Intermediate Monocyte | *ABI3* |
| 1.365905309 | 0.383 | 0.174 | 8.81E-154 | Intermediate Monocyte | *NR4A1* |
| 1.127582234 | 0.387 | 0.173 | 2.40E-147 | Intermediate Monocyte | *TCF7L2* |
| 1.199048739 | 0.378 | 0.187 | 1.39E-124 | Intermediate Monocyte | *MARCKSL1* |
| 1.13152361 | 0.345 | 0.182 | 1.83E-93 | Intermediate Monocyte | *HSPB1* |
| 1.175293982 | 0.261 | 0.126 | 7.51E-81 | Intermediate Monocyte | *ZNF703* |
| 1.158155854 | 0.292 | 0.15 | 8.56E-81 | Intermediate Monocyte | *RUNX3* |
| 5.287892692 | 0.965 | 0.104 | 0 | Nonclassical Monocyte | *FCGR3A* |
| 6.348034555 | 0.852 | 0.036 | 0 | Nonclassical Monocyte | *CDKN1C* |
| 4.972291589 | 0.558 | 0.024 | 0 | Nonclassical Monocyte | *HES4* |
| 6.427807406 | 0.468 | 0.009 | 0 | Nonclassical Monocyte | *CKB* |
| 4.291744272 | 0.409 | 0.025 | 0 | Nonclassical Monocyte | *NEURL1* |
| 4.358036623 | 0.347 | 0.026 | 0 | Nonclassical Monocyte | *C1QA* |
| 4.108397565 | 0.337 | 0.022 | 0 | Nonclassical Monocyte | *ICAM4* |
| 9.526832798 | 0.296 | 0.002 | 0 | Nonclassical Monocyte | *LYPD2* |
| 7.047656738 | 0.297 | 0.003 | 0 | Nonclassical Monocyte | *AC104809.2* |
| 4.600835249 | 0.297 | 0.014 | 0 | Nonclassical Monocyte | *PPM1N* |
| 3.015944702 | 0.711 | 0.175 | 0 | ISG+ Monocyte | *MX1* |
| 2.669994842 | 0.828 | 0.339 | 0 | ISG+ Monocyte | *ISG15* |
| 3.049740135 | 0.501 | 0.086 | 0 | ISG+ Monocyte | *IFI44L* |
| 2.730758965 | 0.444 | 0.1 | 0 | ISG+ Monocyte | *OAS3* |
| 2.576640351 | 0.439 | 0.1 | 0 | ISG+ Monocyte | *IFI44* |
| 3.50293648 | 0.371 | 0.054 | 0 | ISG+ Monocyte | *HERC5* |
| 3.035873685 | 0.348 | 0.067 | 0 | ISG+ Monocyte | *IFIT3* |
| 3.144875768 | 0.339 | 0.071 | 0 | ISG+ Monocyte | *IFIT2* |
| 2.80032697 | 0.286 | 0.055 | 0 | ISG+ Monocyte | *OASL* |
| 2.904341413 | 0.268 | 0.051 | 0 | ISG+ Monocyte | *CMPK2* |
| 4.126001096 | 0.655 | 0.08 | 0 | DC | *CLEC10A* |
| 6.742930912 | 0.582 | 0.013 | 0 | DC | *FCER1A* |
| 5.804439749 | 0.538 | 0.025 | 0 | DC | *CD1C* |
| 3.259006254 | 0.87 | 0.367 | 0 | DC | *HLA-DQA1* |
| 5.544986707 | 0.412 | 0.013 | 0 | DC | *ENHO* |
| 3.230775956 | 0.421 | 0.041 | 0 | DC | *ITGB7* |
| 4.644210883 | 0.377 | 0.029 | 0 | DC | *AREG* |
| 5.081310115 | 0.356 | 0.009 | 0 | DC | *NDRG2* |
| 3.599982864 | 0.346 | 0.024 | 0 | DC | *HLA-DOA* |
| 3.530287737 | 0.297 | 0.016 | 0 | DC | *SLC38A1* |
| 10.53471396 | 0.653 | 0.004 | 0 | pDC | *GZMB* |
| 9.802517765 | 0.572 | 0.002 | 0 | pDC | *CLEC4C* |
| 10.46473179 | 0.524 | 0 | 0 | pDC | *LRRC26* |
| 10.21918894 | 0.486 | 0.001 | 0 | pDC | *SCT* |
| 11.12604439 | 0.465 | 0 | 0 | pDC | *PTPRS* |
| 11.49420468 | 0.368 | 0 | 0 | pDC | *PACSIN1* |
| 12.10738277 | 0.315 | 0 | 0 | pDC | *SHD* |
| 10.90501164 | 0.292 | 0 | 0 | pDC | *MYBL2* |
| 12.65881122 | 0.284 | 0 | 0 | pDC | *AC097375.1* |
| 10.77355549 | 0.281 | 0 | 0 | pDC | *AEBP1* |

| **Table S11. Signature genes used to define functional gene sets.** | |
| --- | --- |
| **signature** | **genes** |
| **Exhaustion score** | *HAVCR2*  *PDCD1*  *PDCD1LG2*  *BHLHE40*  *CD274*  *CDKN1A*  *CR2*  *ATR*  *BAG6*  *BDNF*  *EVI5*  *ID1*  *ID3*  *IL17A*  *IL6*  *IL7R*  *KDM5B*  *LGALS9*  *MAOA*  *PON1* |

| Table S12. Comparison of the AUC among the IgG4 model, 4-variable model, and combined model. | | | |
| --- | --- | --- | --- |
| Model | **AUC** | **CI lower** | **CI upper** |
| IgG4 | 0.871 | 0.753291 | 0.988043 |
| 4-variable | 0.881 | 0.774369 | 0.988297 |
| Combined | 0.927 | 0.832065 | 1 |

area under the receiver operating characteristic curve, AUC; confidence interval, CI

| Table S13. Pairwise comparison of AUCs among the IgG4 model, 4-variable model, and combined model using the DeLong test | |
| --- | --- |
| Comparison | **P value** |
| 4-variable vs IgG4 | 0.851924 |
| Combined vs 4-variable | 0.302704 |
| Combined vs IgG4 | 0.067434 |

area under the receiver operating characteristic curve, AUC;

| Table S14. Net reclassification improvement (NRI) for pairwise comparisons among the IgG4 model, 4-variable model, and combined model | | | |
| --- | --- | --- | --- |
| Comparison | **NRI** | **NRI plus** | **NRI minus** |
| 4-variable vs IgG4 | -0.29 (-0.55, -0.04) | -0.32 (-0.57, -0.07) | 0.03 (0.00, 0.10) |
| Combined vs 4-variable | 0.32 (0.08, 0.56) | 0.32 (0.08, 0.56) | 0.00 (0.00, 0.00) |
| Combined vs IgG4 | 0.11 (0.00, 0.26) | 0.08 (0.00, 0.20) | 0.03 (0.00, 0.11) |

Net reclassification improvement, NRI

| Table S15. Integrated discrimination improvement (IDI) for pairwise comparisons among the IgG4 model, 4-variable model, and combined model | |
| --- | --- |
| Comparison | **IDI** |
| 4-variable vs IgG4 | -0.191 (-0.392, 0.020) |
| Combined vs 4-variable | 0.318 (0.141, 0.494) |
| Combined vs IgG4 | 0.128 (0.066, 0.202) |

Integrated discrimination improvement, IDI

**List of abbreviations**

ABCs: age associated B cells

AIP: Autoimmune pancreatitis

APCs: antigen-presenting cells

AUC: area under the curve

CP: chronic pancreatitis

CTLs: cytotoxic T lymphocytes

CTL-Tfh: cytotoxic Tfh

DCs: dendritic cells

DEGs: differentially expressed genes

ECM: extracellular matrix

GC: germinal center

GO BP: GO biological process

GSVA: Gene Set Variation Analysis

HC: healthy controls

ICDC: International Consensus Diagnostic Criteria

IFN-γ: interferon-γ

IgG4: Immunoglobulin G4

IgG4-RD: IgG4-related disease

IgG4-RI: IgG4 Responder Index

IL-6: interleukin-6

IL-21: interleukin-21

LOXL: lysyl oxidase homologue

MAIT: mucosal-associated invariant T

M-CSF: macrophage colony-stimulating factor

MHC: major histocompatibility complex

PBMC: Peripheral blood mononuclear cells

PCA: principal component analysis

PC/PB: plasma cells / plasmablast

PDAC: pancreatic ductal adenocarcinoma

pDCs: plasmacytoid dendritic cells

PDGF-β: platelet-derived growth factor β

ROC: Receiver operating characteristic

scRNA-seq: single-cell RNA sequencing

TEMRA: terminally differentiated effector memory

Tfh: T follicular helper

Tfr: regulatory follicular helper T

TLR: Toll-like receptor

TME: tumor microenvironment

TNF: tumor necrosis factor

Treg: regulatory T cells

UMAP: uniform manifold approximation and projection
